# CXCR6^+^CD69^+^ CD8^+^ T cells in ascites are associated with disease severity in patients with cirrhosis

**DOI:** 10.1016/j.jhepr.2024.101074

**Published:** 2024-03-24

**Authors:** Christian Niehaus, Sebastian Klein, Benedikt Strunz, Erich Freyer, Benjamin Maasoumy, Heiner Wedemeyer, Niklas K. Björkström, Anke R.M. Kraft, Markus Cornberg

**Affiliations:** 1 Department of Gastroenterology, Hepatology, Infectious Diseases and Endocrinology, Hannover Medical School, Hannover, Germany; 2 Centre for Individualised Infection Medicine (CiiM), a joint venture between the Helmholtz Centre for Infection Research (HZI) and Hannover Medical School (MHH), Hannover, Germany; 3 Twincore, Centre for Experimental and Clinical Infection Research, a joint venture between the Helmholtz Centre for Infection Research (HZI) and the Hannover Medical School, Hannover, Germany; 4 German Center for Infection Research (DZIF), Partner-site Hannover-Braunschweig, Hannover, Germany; 5 Cluster of Excellence RESIST (EXC 2155), Hannover Medical School, Hannover, Germany; 6 CAIMed – Center for AI in Medicine, Joint Venture of Leibniz University Hannover and Hannover Medical School, Hannover, Germany; 7 Center for Infectious Medicine, Department of Medicine Huddinge, Karolinska Institutet, Karolinska University Hospital Huddinge, Stockholm, Sweden; 8 German Center for Infection Research, HepNet Study-House German Liver Foundation, Hannover, Germany

**Keywords:** Acute-on-chronic liver failure, Bystander activation, JAK inhibitor, Peritoneal cavity, Tissue residency

## Abstract

**Background & Aims:**

Patients with advanced cirrhosis often develop hepatic decompensation, which is accompanied by systemic inflammation and may eventually lead to acute-on-chronic liver failure. One important cause of systemic hyperinflammation is a dysregulated overshooting immune response in ascites in the abdominal cavity. In this study, we analyzed the role of CD8^+^ T cells in the ascites immune compartment.

**Methods:**

Peripheral blood and ascites fluid were collected from 50 patients with decompensated cirrhosis. Phenotype and functional responses of CD8^+^ T cells were analyzed, and obtained data were compared with each other as well as with healthy controls and patients with compensated cirrhosis.

**Results:**

High-dimensional flow cytometry revealed that CD8^+^ T cells are abundant in the ascites of patients with cirrhosis and exhibit a chronically activated bystander phenotype with innate-like functions. Indeed, we identified distinct CXCR6^+^CD69^+^ clusters of late effector memory CD8^+^ T cells that were rarely found in blood and correlated with clinical parameters of disease severity. Moreover, this CD8^+^ T-cell population was hyperresponsive to innate cytokines and exhibited cytokine-mediated bystander activation. Interestingly, the Janus kinase (JAK) inhibitor tofacitinib was able to effectively block bystander-activated CXCR6^+^CD69^+^ CD8^+^ T cells and significantly suppress effector molecule production.

**Conclusions:**

The results indicate that CXCR6^+^CD69^+^ CD8^+^ T cells in ascites are associated with disease severity and may contribute to inflammation in patients with decompensated cirrhosis, suggesting that targeted inhibition of this immune cell subset may be a viable therapeutic option.

**Impact and Implications:**

Patients with advanced cirrhosis often develop hepatic decompensation, which is accompanied by systemic inflammation and eventually leads to acute-on-chronic liver failure. One important cause of systemic hyperinflammation is a dysregulated overshooting immune response in ascites in the abdominal cavity. In this study, we demonstrate that CXCR6^+^CD69^+^ CD8^+^ T cells are abundant in the ascites of patients with cirrhosis, exhibit a chronically activated bystander phenotype, and correlate with clinical parameters of disease severity. Moreover, we show that the Janus kinase (JAK) inhibitor tofacitinib can effectively block these bystander-activated CXCR6^+^CD69^+^ CD8^+^ T cells, suggesting that targeted inhibition of this immune cell subset may be a potential therapeutic strategy.

**Clinical trial number:**

Prospective registry: INFEKTA (DRKS00010664).

## Highlights

• CD8^+^ T cells are abundant in the ascites of patients with cirrhosis and exhibit an activated bystander phenotype.
• Co-expression of CXCR6 and CD69 identifies distinct clusters of CD8^+^ T cells that correlate with liver disease severity.
• CXCR6^+^CD69^+^ CD8^+^ T cells are hyperresponsive to innate cytokines and exhibit cytokine-mediated bystander activation.
• The JAK inhibitor tofacitinib can effectively block bystander-activated CXCR6^+^CD69^+^ CD8^+^ T cells.

## Graphical abstract

**Figure:**
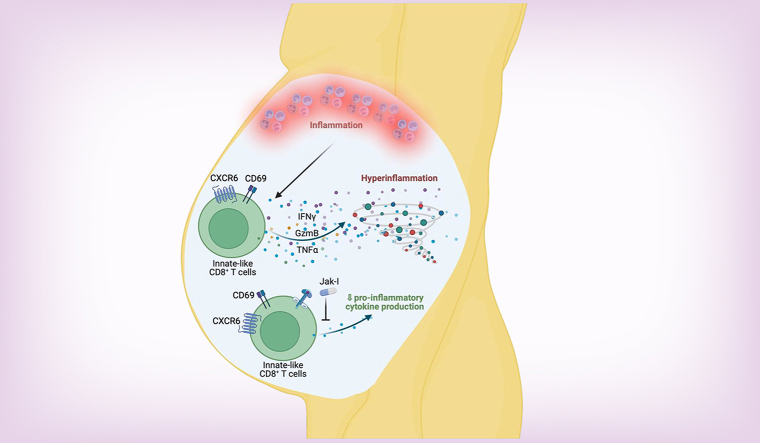

## Introduction

Cirrhosis is the result of persistent liver damage and is commonly caused by chronic liver diseases such as chronic viral hepatitis, alcoholic liver disease, and non-alcohol steatohepatitis. In the general worldwide population, the prevalence of cirrhosis is between 0.2 and 0.3%, and more than 1 million deaths per year are attributed to complications of cirrhosis.[1], [2], [3] Patients with advanced cirrhosis often develop hepatic decompensation, which is accompanied by systemic inflammation and may ultimately result in acute-on-chronic liver failure (ACLF) and death.[4], [5], [6] The decompensated state of cirrhosis is marked by the development of apparent clinical signs, the most frequent of which are ascites, hepatic encephalopathy, esophageal varices bleeding, and the occurrence of icterus.^7^^,^^8^

Patients with cirrhosis have a higher susceptibility to bacterial infections, and one reason for the vulnerability to infections is meant to be the cirrhosis-associated immune deficiency (CAID) syndrome.^9^^,^^10^ CAID involves systemic immune dysfunction and leads to a state of systemic inflammation, driven by persistent immune cell activation, which can result in an overshooting immune response with increased production of pro-inflammatory cytokines and upregulated expression of cell activation markers.^9^^,^^11^

One lymphocyte subset previously shown to contribute to the state of systemic inflammation and subsequently play a critical role in patients with liver disease is CD8^+^ T cells.[11], [12], [13], [14], [15], [16], [17], [18]

On the one hand, CD8^+^ T cells are key participants in the adaptive immune response, requiring T-cell receptor (TCR)-mediated stimulation by binding cognate peptide presented by major histocompatibility complex class 1 (MHC-1) to provide rapid protection against previously encountered pathogens and to perform immunomodulatory functions.[19], [20], [21], [22] On the other hand, T-cell activation without cognate antigens often relies on non-specific extrinsic factors, including cytokines, and this TCR-independent activation can drive immune-mediated pathogenesis, particularly in chronic diseases.^14^^,^^20^^,^^23^

In more detail, recent research suggests a dysregulated, non-specific activation of CD8^+^ T cells leading to immune-mediated liver pathology.[12], [13], [14], [15], [16], [17] In patients with cirrhosis, peripheral blood CD8^+^ T cells are activated and correlated with disease progression.^11^ In addition, CD8^+^ T cells from the liver of patients with HDV infection show an antigen-non-specific activation of liver-resident CD8^+^ T cells, which contributes to the disease stage.^13^ This is also in line with previous studies showing an accumulation of activated T cells in patients with different liver diseases, subsequently resulting in liver damage.^12^^,^^14^^,^^15^^,^^24^ Furthermore, Dudek *et al.*^12^ and Nkongolo *et al.*^14^ recently demonstrated that CD8^+^ T cells expressing the tissue-residency marker CXCR6 have direct hepatotoxic potential and contribute to liver immune pathology through an innate-like, auto-aggressive killing activity, as well as by driving severe inflammation.

Having previously shown that the ascites of patients with decompensated cirrhosis harbor a soluble inflammatory environment,^25^ we used these novel findings as a rationale to analyze the ascites CD8^+^ T-cell compartment in more detail. We hypothesize that in patients with decompensated cirrhosis and ascites, bystander T-cell activation is existent.

## Materials and methods

### Study design and sample collection

Fifty patients with cirrhosis and ascitic decompensation without spontaneous bacterial peritonitis (SBP) were recruited in the prospective registry INFEKTA (DRKS00010664). When available, matched samples (n = 43) of peripheral blood (peripheral blood mononuclear cells [PBMCs] and plasma) and ascites (mononuclear cells [MNCs] and supernatant) were obtained from these patients. In patients with compensated cirrhosis, only peripheral blood (PBMCs and plasma) was collected. All patients were seen at the Department of Gastroenterology, Hepatology, Infectious Diseases and Endocrinology at Hannover Medical School. The diagnosis of cirrhosis was based on clinical, radiological, or histological findings. ACLF was diagnosed according to the European Association for the Study of the Liver–Chronic Liver Failure (EASL-CLIF) Consortium criteria.^26^^,^^27^ Only patients without the presence of SBP, which was diagnosed in patients with ≥250 polymorphonuclear cells/mm^3^ ascites fluid,^28^ were considered for inclusion. Exclusion criteria for this study were hepatocellular carcinoma and/or HIV infection. In addition, peripheral blood from healthy volunteers was collected as controls. Detailed patient characteristics are presented in Table S1.

### Isolation and storage of PBMCs, plasma, and MNCs from ascites, and ascites supernatant

Peripheral blood was collected during daily routine blood sampling, and ascites was collected during paracentesis as indicated by a physician. Subsequently, PBMCs and MNCs were isolated from fresh whole blood using Ficoll density gradient centrifugation and were then cryopreserved in liquid nitrogen according to the standard operating procedures of the Hannover Unified Biobank. Plasma and ascites supernatant were collected from EDTA blood and ascites samples via centrifugation, as previously described,^25^ and stored at -80 °C.

### Flow cytometric analysis of PBMCs and MNCs

Cryopreserved PBMCs and ascites MNCs were thawed, and flow cytometry staining with fluorochrome-labeled monoclonal antibodies was performed, as previously described.^29^ For dead cell exclusion, all samples were stained with Fixable Viability Stain 700, Live Dead Fixable Aqua, or Live Dead Fixable Green. For fixation, eBioscience Fixative (FOXP3/Transcription Buffer Staining Set, eBioscience) was used. Identification of ɣδ T cells, mucosa-associated invariant T (MAIT) cells, and natural killer T (NKT) cells among CXCR6^+^CD69^+^ CD8^+^ T cells was performed by surface staining for TCR ɣδ, CD161 and TCR Va7.2, and CD161 and CD56, respectively, as previously described.^30^

Samples were acquired on either a 16- or 18-color LSR Fortessa flow cytometer (BD Biosciences) as well as a 30-color BD symphony flow cytometer (BD Biosciences). The obtained data were analyzed by conventional flow cytometry using FlowJo software v10.5.3 (BD Biosciences). For high-dimensional analysis, Uniform Manifold Approximation and Projection (UMAP) and PhenoGraph analysis were performed using the open-access available FlowJo plugins.^31^^,^^32^

### Functional assays of PBMCs and MNCs

PBMCs and ascites MNCs were stimulated with different cytokine combinations for 24 h, as previously described.^25^ In brief, PBMCs and ascites MNCs were stimulated with either IL-12 (10 ng/ml) + IL-18 (100 ng/ml) or IL-6 (10 ng/ml) + IL-21 (10 ng/ml) to induce cytokine response. PMA/ionomycin (2 μg/ml; 500 ng/ml) was used as a positive control. For the last 6 h of stimulation, brefeldin A (2 μg/ml; Golgi Plug, BD Biosciences) and monensin (Golgi Stop, BD Biosciences), as well as CD107a, to assess degranulation were added. In selected experiments, PBMCs were either stimulated for 5 days with IL-15 (10 ng/ml) with 200 μl media/well, or with ascites supernatant (100 μl media + 100 μl ascites supernatant), or left untreated.

### *In vitro* treatment with tofacitinib

In selected experiments, the JAK inhibitor tofacitinib (10 or 20 μM; Sigma Aldrich) was added to the functional assays and incubated for 2 h at 37 °C before stimulation with IL-12 + IL-18.

### Cytokine assays and correlations

Cytokine levels of plasma and ascites supernatant were previously measured using the LUMINEX-based multiplex bead assay (Human Cytokine Assay; 12007283; Bio-Rad, USA) according to the manufacturer’s instructions and optimized protocols.^25^ All samples were analyzed in one run and acquired using BioPlex Manager 6.0 software. Cytokine values were correlated with frequencies of CXCR6^+^CD69^+^ CD8^+^ T cells when indicated.

### Single-cell RNA sequencing

Single-cell transcript libraries and the respective TCR complementarity determining region 3 were prepared using a targeted approach of the BD Rhapsody Single-Cell Express System (BD Biosciences) following the manufacturer’s guidelines. Libraries were sequenced using an Illumina NextSeq platform (Illumina, San Diego, CA, USA) in a 225/75-bp paired-end mode.

The obtained RNA-sequencing data were processed using a cwl-tool via the BD Rhapsody Analysis Pipeline (v1.9) on a local server. After quality control, normalization, and batch correction, the R package “Seurat” (v4; R Foundation for Statistical Computing, Vienna, Austria) was used (e.g. UMAP and FindAllMarkers).^33^ Further analysis was conducted using the Python-based packages scanpy^34^ and scirpy (Python Software Foundation, Wilmington, DE, USA).^35^

### Statistical analysis

Statistical analyses were performed using GraphPad Prism software 9.0 (GraphPad Software, San Diego, CA, USA). First, the distribution of datasets was assessed using the D’Agostino–Pearson normality test. For unmatched and normally distributed data, an unpaired *t* test was used to compare between two datasets, whereas for datasets not normally distributed, the Mann–Whitney *U* test was performed. Matched samples were analyzed using either the Wilcoxon matched-pairs signed-rank test or a paired *t* test, depending on the normal distribution of the data. For multiple comparisons, the Kruskal–Wallis test or ANOVA was used when indicated. Correlations between datasets were analyzed using either Person’s *r* or Spearman’s *r* coefficients, depending on the distribution of datasets. Details regarding statistical tests are displayed in the figure legends, and for all graphs, significances are indicated as ∗*p* <0.05, ∗∗*p* <0.01, ∗∗∗*p* <0.001, and ∗∗∗∗*p* <0.0001.

### Study approval

Written informed consent was obtained from all participants before inclusion. The study was approved by the Ethics Committee of Hannover Medical School (3188-2016), and the study protocol conforms to the ethical guidelines of the 1975 Declaration of Helsinki.

## Results

### CD8^+^ T cells are decreased in peripheral blood of patients with decompensated cirrhosis but enriched in the peritoneal cavity

Cirrhosis affects immune cells both in peripheral blood and locally.^9^ One of the most frequent consequences of hepatic decompensation is the accumulation of a substantial amount of ascites in the peritoneal cavity. In this study, we investigated the role of T cells in the immune compartment ascites. A total of 60 patients with cirrhosis and 25 healthy controls were included. In more detail, 50 patients had decompensation of cirrhosis as an occurrence of ascites. From these patients, matched peripheral blood and ascites fluid were collected. All patients included in this study had no evidence of SBP. From the remaining 10 patients, who had compensated cirrhosis, blood samples were collected. Detailed patient baseline characteristics are presented in Table S1.

As circulating T cells have previously been shown to contribute to systemic inflammation and play a critical role in patients with cirrhosis,^11^^,^^18^ in this study, we examined the CD4^+^ as well as CD8^+^ T-cell compartment in detail, focusing on the peritoneal cavity. The full gating strategy to identify total T cells and CD4^+^ as well as CD8^+^ T cells is displayed in Fig. S1A.

**Fig. 1:**
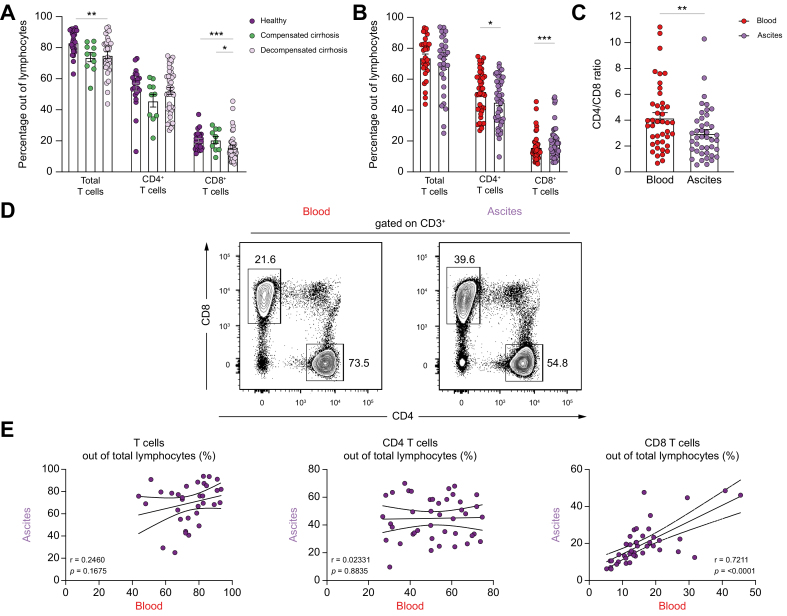
CD8^+^ T cells are decreased in peripheral blood of patients with decompensated cirrhosis but enriched in the peritoneal cavity. (A) Frequencies of total T cells, CD4^+^ T cells, and CD8^+^ T cells out of total lymphocytes in patients with compensated (n = 10) and decompensated (n = 43) cirrhosis compared with healthy controls (n = 24). (B) Frequencies of total T cells, CD4^+^ T cells, and CD8^+^ T cells out of total lymphocytes in the ascites of patients with decompensated cirrhosis compared with matched peripheral blood samples (n = 43). (C) CD4/CD8 T-cell ratio in the ascites compared with peripheral blood of matched patients (n = 43). (D) Representative FACS plots showing a CD4^+^ and CD8^+^ T-cell staining from matched blood and ascites samples from one patient with decompensated cirrhosis. (E) Correlations of frequencies of peripheral blood total T cells, CD4^+^ T cells, and CD8^+^ T cells out of total lymphocytes with ascites T cell subsets. The Mann–Whitney *U* test and an unpaired *t* test, as well as the Wilcoxon test or a paired *t* test, were used when appropriate, and correlations were assumed using Spearman’s *r* for all non-parametric values. ∗*p* <0.05; ∗∗*p* <0.01; ∗∗∗*p* <0.001; ∗∗∗∗*p* <0.0001.

In line with previous reports,^11^^,^^18^ we observed significantly lower frequencies of total blood T cells in patients with decompensated cirrhosis than in healthy volunteers. This decrease was attributed to decreasing frequencies of CD8^+^ T cells, whereas CD4^+^ T-cell frequencies remained stable (Fig. 1A). Because of the observed decline of circulating T-cell frequencies with cirrhosis decompensation, we investigated these lymphocyte subsets in more detail in patients with advanced cirrhosis and especially focus on the peritoneal cavity. Interestingly, we did not observe a difference in the frequency of total T cells in ascites samples compared with matched blood samples. However, we noticed a significant decrease in the CD4^+^ T-cell compartment, whereas CD8^+^ T-cell frequencies were significantly increased (Fig. 1B and D). This was further corroborated by a decline in the CD4/CD8 T-cell ratio, indicating a shift towards CD8^+^ T cells within the ascites T-cell compartment (Fig. 1C). Interestingly, we found a strong positive correlation between frequencies of blood CD8^+^ T cells and matched ascites CD8^+^ T cells, whereas there were no correlations for CD4^+^ T cells and total T cells (Fig. 1E and Fig. S6A).

Taken together, CD8^+^ T cells are decreased in the peripheral blood of patients with decompensated cirrhosis but are enriched in the peritoneal cavity.

### CD8^+^ T cells in the peritoneal cavity express an activated and exhausted memory-like phenotype

**Fig. 2:**
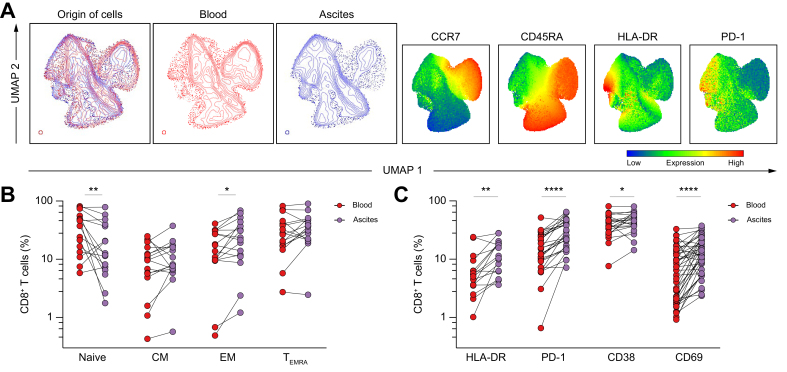
CD8^+^ T cells in the peritoneal cavity express an activated and exhausted memory-like phenotype. (A) UMAP analysis was performed on four surface markers for 30 concatenated matched ascites and blood samples with 10,000 live CD45^+^CD3^+^CD8^+^ cells from each sample. UMAP plots from the ascites fluid (blue) of patients with decompensated cirrhosis and their respective blood (red) samples showing the relative abundance of each group and UMAP plots for each of the four markers are displayed. (B) Frequencies of naïve, CM, EM, and T_EMRA_ cells out of CD8^+^ T cells (n = 16) and (C) expression of activation and exhaustion markers on CD8^+^ T cells in blood compared with matched ascites (n = 16–43). The Wilcoxon test or a paired *t* test was used when appropriate. ∗*p* <0.05; ∗∗*p* <0.01; ∗∗∗*p* <0.001; ∗∗∗∗*p* <0.0001. CM, central memory; EM, effector memory; T_EMRA_, terminally differentiated effector memory; UMAP, Uniform Manifold Approximation and Projection.

Given the higher frequency of CD8^+^ T cells in ascites, it is of interest to characterize this enrichment. Therefore, we next investigated the phenotype of ascites CD8^+^ T cells in more detail. UMAP analysis and manual gating revealed several differences in differentiation and activation between the different compartments (Fig. 2A–C). In more detail, the expression of PD-1 and HLA-DR was higher in ascites CD8^+^ T cell clusters, and the lack of CD45RA was observed in these ascites clusters, demonstrating a shift from circulating naïve CD8^+^ T cells toward central memory and effector memory cells in the ascites (Fig. 2A and B). In line with this, the activation markers CD38 and CD69 were also upregulated on ascites CD8^+^ T cells (Fig. 2C). The activated phenotype of ascites CD8^+^ T cells is consistent with a previous report.^11^ Interestingly, we observed a higher abundance of heterogenous subsets of primarily less differentiated but activated PD-1^+^CD127^+^ memory-like cells, as well as further differentiated and more severely exhausted PD-1^+^CD127^-^ cells within peritoneal CD8^+^ T cells compared with those in circulation (Fig. S2A). Consistent with this, most circulating CD8^+^ T cells exhibited a PD-1^-^CD38^+^ or PD-1^-^CD38^-^ phenotype, whereas CD8^+^ T cells derived from the peritoneal cavity displayed a highly activated and exhausted PD-1^+^CD38^+^ or PD-1^+^CD38^-^ phenotype (Fig. S2B).

Taken together, these results indicate that CD8^+^ T cells in the peritoneal cavity of patients with decompensated cirrhosis express an activated and exhausted memory-like phenotype compared with peripheral blood CD8^+^ T cells. These data are consistent with a previous report^11^ and validate our cohort for downstream analysis.

### UMAP analysis reveals a specific cluster of CD8^+^ T cells in ascites with a late effector memory phenotype and increased expression of tissue retention markers

**Fig. 3:**
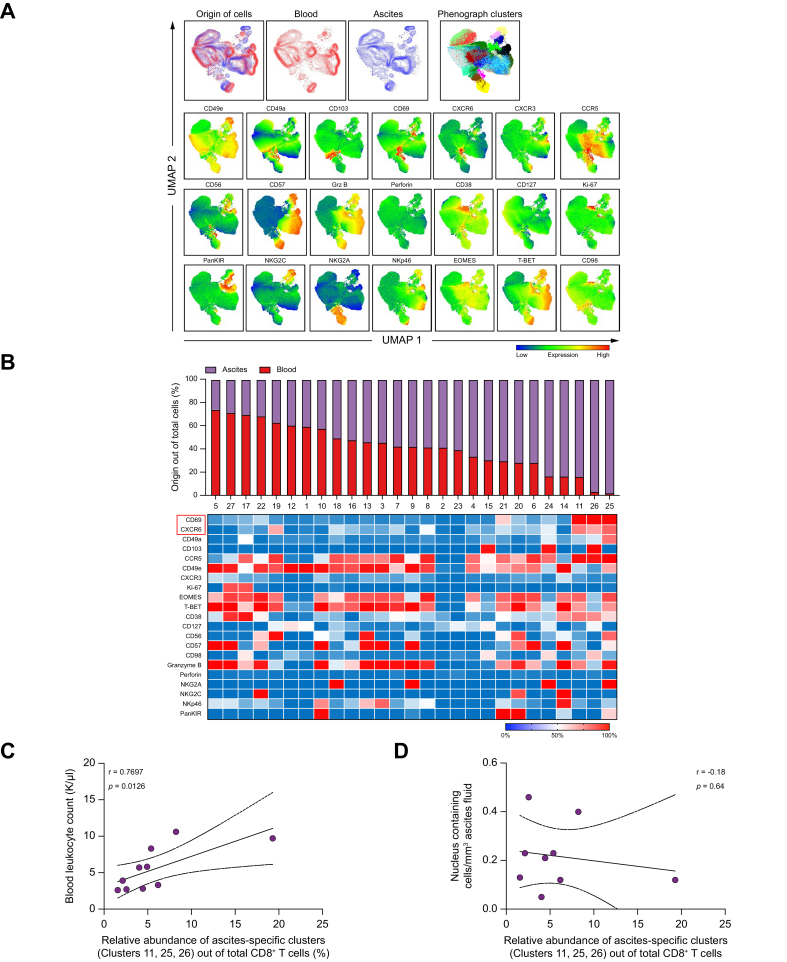
UMAP analysis reveals a specific cluster of CD8^+^ T cells in ascites with a late effector memory phenotype and increased expression of tissue retention markers. (A) UMAP plots on the gated CD8^+^ T-cell population, displaying the different origin (blood and ascites) of CD8^+^ T cells and respective PhenoGraph clusters. UMAP analysis was performed on 21 markers for 10 matched ascites and blood samples with 10,000 CD8^+^ T cells exported from each sample. Single marker plots displaying the main phenotypic clusters, their relative abundance in ascites compared with blood, and the markers included in the analysis. (B) PhenoGraph analysis identifying 27 different clusters of CD8^+^ T cells and the relative abundance of each of the identified clusters comparing blood and ascites, with the deconvoluted phenotype of each cluster displayed as a heatmap. (C, D) Correlation of the relative abundance of the three ascites-specific clusters (11, 25, and 26) with blood leukocyte count (C) and nucleus containing cells/mm^3^ ascites fluid (D). Correlations were assumed using Spearman’s *r* for all non-parametric values. ∗*p* <0.05; ∗∗*p* <0.01. UMAP, Uniform Manifold Approximation and Projection.

Having established that peritoneal CD8^+^ T cells are activated and display a memory-like phenotype, we next performed combined UMAP and PhenoGraph analysis to identify unique and shared clusters of CD8^+^ T cells derived from ascites and matched peripheral blood. To this end, the expression of 21 phenotypic markers was analyzed, and 27 clusters could be identified through PhenoGraph analysis when comparing peripheral blood and ascites CD8^+^ T cells (Fig. 3A and B).

Ascites CD8^+^ T cells expressed higher levels of the tissue-residency markers CD103, CD49a, CD69, CXCR6, and CCR5 compared with blood CD8^+^ T cells (Fig. 3A), and clustering was largely based on these tissue-resident markers (Fig. 3B). In addition, these observations were also corroborated by manual gating (Fig. S3B–E). Of note, circulating CD8^+^ T cells expressed higher levels of the transcription factor T-bet, whereas Eomes expression was similar to that in ascites CD8^+^ T cells (Fig. 3A and Fig. S3A). Moreover, CD127 (IL-7Rα) was downregulated on peritoneal CD8^+^ T cells, indicating previous immune activation and TCR stimulation,^14^ and this was also paralleled by diminished proliferative capacity (Fig. S3A). Interestingly, the inhibitory natural killer (NK) cell receptor NKG2A was also upregulated on ascites CD8^+^ T cells, suggesting strong T-cell activation and cytotoxicity^36^ (Fig. S3A).

Intriguingly, we could identify three distinct clusters of ascites CD8^+^ T cells that were very rarely found in peripheral blood. The defining markers for these ascites-specific clusters were co-expression of CD69 and CXCR6 being absent on CD8^+^ T cells from blood (clusters 11, 26, and 25) (Fig. 3B). Furthermore, these clusters expressed high levels of the activation marker CD38 and the tissue-residency marker CCR5 (Fig. 3A and B). Moreover, the relative abundance of the ascites-specific clusters within the CD8^+^ T-cell compartment (clusters 11, 26, and 25) was further correlated with blood leukocyte count, but not with nucleus-containing cells in the ascites fluid (Fig. 3C and D).

To conclude, these results suggest that the ascites of patients with decompensated cirrhosis harbor certain CD8^+^ late effector memory T cell clusters with increased expression of tissue retention markers.

### Ascites CXCR6^+^CD69^+^ CD8^+^ T cells correlate with disease severity and express markers of innate-like bystander activation

**Fig. 4:**
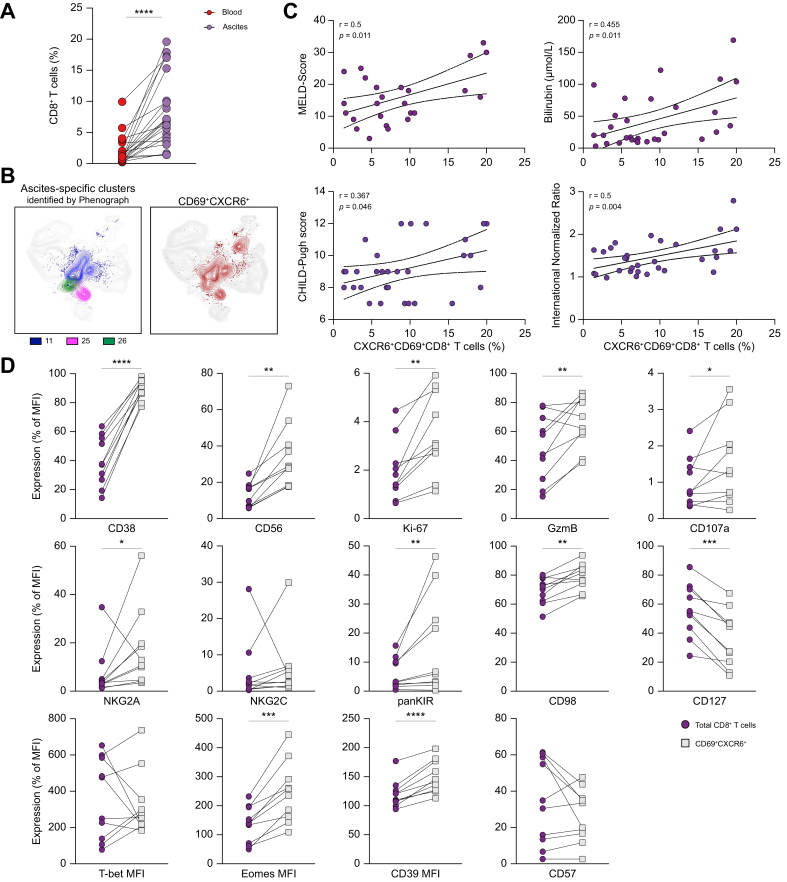
Ascites CXCR6^+^CD69^+^ CD8^+^ T cells correlate with disease severity and express markers of innate-like bystander activation. (A) Frequencies of CXCR6^+^CD69^+^ CD8^+^ T cells in matched blood and ascites (n = 20). (B) Ascites-specific clusters identified by PhenoGraph analysis (clusters 11, 25, and 26) in comparison with co-expression of CXCR6 and CD69. (C) Correlations of frequencies of CXCR6^+^CD69^+^ CD8^+^ T cells with clinical markers of liver disease severity (n = 30–31). (D) Comparison of phenotypic markers on peritoneal total CD8^+^ T cells compared with CXCR6^+^CD69^+^ CD8^+^ T cells (n = 10). The Wilcoxon test or a paired *t* test was used when appropriate. Correlations were assumed using Spearman r coefficients for non-parametric datasets. ∗*p* <0.05; ∗∗*p* <0.01; ∗∗∗*p* <0.001; ∗∗∗∗*p* <0.0001. MFI, mean fluorescence intensity; MELD, model for end-stage liver disease.

As we observed that co-expression of CD69 and CXCR6 defined distinct clusters in the ascites, we next sought to analyze the phenotype of ascites CXCR6^+^CD69^+^ CD8^+^ T cells in more detail. As expected, CXCR6^+^CD69^+^ CD8^+^ T cells were more abundant in the ascites fluid than in matched peripheral blood (Fig. 4A). Along the same lines, we observed extensive overlap of the three ascites-specific clusters (clusters 11, 26, and 25) identified by PhenoGraph and co-expression of CXCR6 and CD69 on CD8^+^ T cells (Fig. 4B). Remarkably, the frequencies of ascites CXCR6^+^CD69^+^ CD8^+^ T cells were significantly correlated with markers of liver disease severity, as indicated by the model for end-stage liver disease score, Child–Pugh score, bilirubin blood levels, and international normalized ratio (Fig. 4C). Of note, we observed a higher proportion of ascites CXCR6^+^CD69^+^ CD8^+^ T cells in patients with ACLF than in patients without ACLF (15.77 *vs*. 8.24%, *p* = 0.029) (Fig. S1B).

Indeed, when comparing CXCR6^+^CD69^+^ CD8^+^ T cells in ascites with total T cells, we observed a higher frequency of CD38, CD56, NKG2A, panKIR, Eomes, and CD39, reflecting a chronically activated phenotype that exhibits features of NK cells that have been previously shown to be associated with interferon γ (IFNγ) production in response to innate stimulation (Fig. 4D).^37^ Of note, Ki-67 expression was upregulated in ascites CXCR6^+^CD69^+^ CD8^+^ T cells compared with total CD8^+^ T cells (Fig. 4D). Moreover, ascites CXCR6^+^CD69^+^ CD8^+^ T cells expressed significantly higher levels of granzyme B (GzmB) and CD98, indicative of bystander activation (Fig. 4D).^20^ Thus, some of the phenotypic differences we observed when comparing circulating and total peritoneal CD8^+^ T cells (Fig. 2, Fig. 3) could be ascribed to the distinctive phenotype of the CXCR6^+^CD69^+^ CD8^+^ T cells.

Altogether, CXCR6^+^CD69^+^ CD8^+^ T cells are chronically activated and exhibit markers of innate-like bystander activation. Furthermore, this cell subset correlates with liver disease severity, which may indicate a pathogenic role of this subset in patients with decompensated cirrhosis and ascites.

### Co-expression of CD69 and CXCR6 defines a transcriptionally activated subset with features of tissue residency and inflammation

**Fig. 5:**
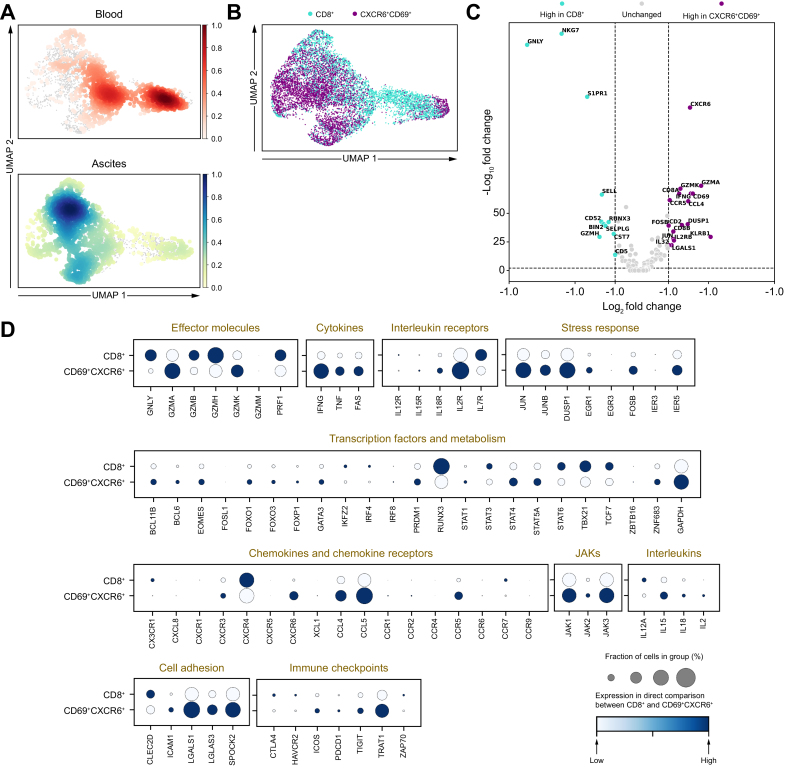
Co-expression of CD69 and CXCR6 defines a transcriptionally activated subset with features of tissue residency and inflammation. (A) UMAP analysis of matched blood and ascites CD8^+^ T cells. (B) Same UMAP of the CD8^+^ T-cell compartment showing differential clustering of CXCR6^+^CD69^+^ CD8^+^ T cells and their respective CD8^+^ T cell counterparts. (C) Volcano plot depicting differentially expressed genes in CXCR6^+^CD69^+^ CD8^+^ T cells compared with their CD8^+^ counterparts. The average log fold change and average Benjamini–Hochberg-corrected *p* values (FDR) for pairwise differential expression are shown. (D) Relative expression of individual genes between CXCR6^+^CD69^+^ CD8^+^ and CXCR6^-^CD69^-^ CD8^+^ T cells. FDR, false discovery rate; Gzm, granzyme; JAK, Janus kinase; TNF, tumor necrosis factor; UMAP, Uniform Manifold Approximation and Projection.

Based on the phenotype analysis, we examined the transcriptional profile of CXCR6^+^CD69^+^ CD8^+^ T cells to validate our previous results generated from flow cytometry analysis. Therefore, we performed single-cell transcriptomics from sorted blood and ascites CXCR6^+^CD69^+^ CD8^+^ T cells and their respective CXCR6^-^CD69^-^ counterparts (Fig. 5). In line with our previous results, we observed a distinct clustering of blood and ascites CD8^+^ T cells (Fig. 5A), and we could further identify a differential clustering of CXCR6^+^CD69^+^ CD8^+^ T cells within the CD8^+^ T-cell compartment (Fig. 5B). Moreover, transcriptional analysis revealed certain significantly differentially expressed genes between paired CXCR6^+^CD69^+^ CD8^+^ T cells and CXCR6^-^CD69^-^ CD8^+^ T cells in ascites (Fig. 5C). In more detail, we found a downregulation of S1PR1 and SELL in CXCR6^+^CD69^+^ CD8^+^ T cells compared with CXCR6^-^CD69^-^ CD8^+^ T cells, which was previously shown to be required for tissue residency,^38^^,^^39^ and therefore indicating features of tissue residency (Fig. 5C). In line with this, we observed an upregulation of CCR5 in CXCR6^+^CD69^+^ CD8^+^ T cells, which is associated with tissue retention and homing, as well as with bystander activation^20^ (Fig. 5C). Furthermore, CCL4, JUN, IL-32, and FOS, which are known to be relevant genes for inflammation,^40^ were upregulated on CXCR6^+^CD69^+^ CD8^+^ T cells compared with their CD8^+^ T-cell counterparts (Fig. 5C). Moreover, IL2RB/IL-15RB was enriched, whereas CD5 was downregulated on CXCR6^+^CD69^+^ CD8^+^ T cells (Fig. 5B and C). Together with higher expression levels of IFNγ, GzmA, and GzmK, this indicates bystander activation.^20^

To obtain a more detailed view of both T cell subsets, a direct heatmap comparison of genes was performed, each assigned to a corresponding group (Fig. 5D). Interestingly, transcriptomic data revealed consistently more JAK–STAT (JAK1/2/3 and STAT1/4/5A) pathway activation and subsequent IFN, tumor necrosis factor (TNF), and FAS transcription in CXCR6^+^CD69^+^ CD8^+^ T cells compared with their CD8^+^ T cell counterparts (Fig. 5D). In line with this, we further observed an upregulation of genes involved in effector function (GZMA/K) as well as several genes involved in stress responses (JUN, JUNB, DUSP1, EGR1, FOSB, and IER5) in CXCR6^+^CD69^+^ CD8^+^ T cells compared with their CD8^+^ T-cell counterparts (Fig. 5D). Similarly, transcriptomic analysis also revealed an upregulation of chemokines and chemokine receptors as well as cell adhesion genes (Fig. 5D). Of note, an upregulation of immune checkpoints (ICOS, PDCD1, TIGIT, and TRAT1) was observed in the CXCR6^+^CD69^+^ subset compared with CD8^+^ T cells (Fig. 5D). Moreover, when comparing the transcriptional differences of these cells in blood and ascites individually, we observed that CXCR6^+^CD69^+^ CD8^+^ T cells are even more distinct between their respective blood and ascites origin compared with their CD8^+^ counterparts (Fig. S7A).

In addition to the transcriptomics, we determined the TCR CDR3 region of CXCR6^+^CD69^+^ CD8^+^ T cells and counterparts and associated all T cells with their respective clonotype. As expected, TCR diversity was comparable between both groups, indicating a clear separation of CXCR6^+^CD69^+^ CD8^+^ T cells from most other innate-like T cells, such as MAIT cells (Fig. S7B). Although almost all clonotypes were private and not shared between patients (Fig. S7C), we found that over 53% appeared in the CXCR6^+^CD69^+^ CD8^+^ and CXCR6^-^CD69^-^ compartments of the patients (Fig. S7D).

Taken together, CXCR6^+^CD69^+^ CD8^+^ T cells display activated transcriptional signatures with features of tissue residency, and TCR analysis indicates a TCR-independent development of the CXCR6^+^CD69^+^ CD8^+^ T-cell subset.

### Peritoneal CD8^+^ T cells are hyperresponsive to innate cytokine stimulation with IL-12 + IL-18

**Fig. 6:**
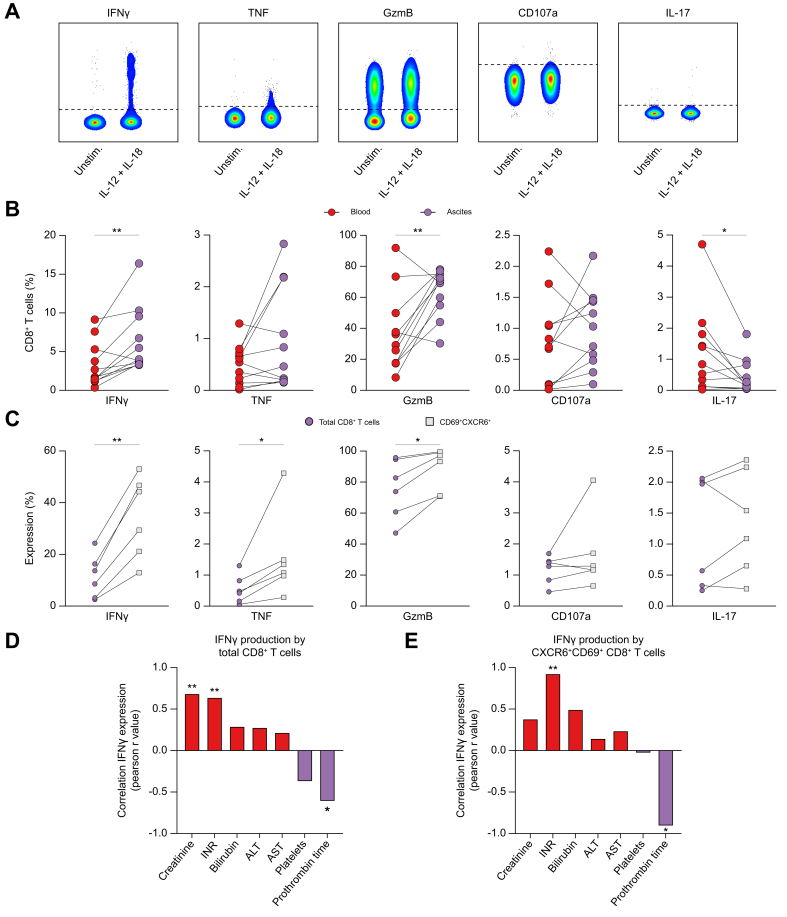
Peritoneal CD8^+^ T cells are hyperresponsive to innate cytokine stimulation with IL-12 + IL-18. (A) Representative concatenated FACS plots showing peritoneal CD8^+^ T cell responses after stimulation with IL-12 + IL-18 compared with unstimulated medium controls. (B) Readouts of functional responses following IL-12 + IL-18 stimulation as indicated by expression of pro-inflammatory cytokines and effector molecules (n = 11). (C) Comparison of functional responses of total CD8^+^ T cells compared with CXCR6^+^CD69^+^ CD8^+^ T cells in the ascites (n = 6). (D, E) Correlations of (D) IFNγ-producing CD8^+^ T cells (n = 11) and (E) IFNγ-producing CXCR6^+^CD69^+^ CD8^+^ T cells (n = 6) in the ascites with markers of disease severity (positive correlations are colored in red and negative correlations in blue). Functions of blood and ascites samples were compared using a paired *t* test or the Wilcoxon test when appropriate. Spearman`*r* was used to correlate among non-parametric data. ∗*p* <0.05; ∗∗*p* <0.01. ALT, alanine aminotransferase; AST, aspartate aminotransferase; GzmB, granzyme B; IFNγ, interferon γ; INR, international normalized ratio; TNF, tumor necrosis factor.

We next assessed the functional capacity of peritoneal CD8^+^ T cells in comparison with matched peripheral blood CD8^+^ T cells upon stimulation with the pro-inflammatory cytokines IL-12 and IL-18. Indeed, the combination of cytokines (IL-12 + IL-18) was able to effectively stimulate CD8^+^ T cells (Fig. 6A and Fig. S4A). Strikingly, ascites CD8^+^ T cells produced higher levels of IFNγ and GzmB as compared with circulating CD8^+^ T cells (Fig. 6B). In contrast, IL-17 was significantly lower on ascites CD8^+^ T cells (Fig. 6B). Having previously shown that MAIT cells, a subset of innate-like T cells, are effective producers of effector molecules upon stimulation with IL-12 + IL-18,^25^ we herein demonstrate that nearly equal levels of effector molecules were produced after exclusion of MAIT cells, indicating that MAIT cells account for only a small proportion of effector molecules produced by total CD8^+^ T cells (Fig. S4C). Consistent with this, the frequencies of ɣδ T cells, MAIT cells, and NKT cells were very low among CXCR6^+^CD69^+^ CD8^+^ T cells (Fig. S4B).

In addition, we observed that the ascites-specific CXCR6^+^CD69^+^ CD8^+^ T-cell subset exhibited higher responsiveness to exogenous IL-12 and IL-18 compared with the entire CD8^+^ T-cell compartment, as indicated by the higher production of IFNγ, TNF, and GzmB, which accounts for the high degree of bystander activation (Fig. 6C). Of note, IFNγ production by ascites CD8^+^ T cells correlated with markers of liver disease severity (Fig. 6D), and this was also true for the CXCR6^+^CD69^+^ CD8^+^ T-cell subset (Fig. 6E).

To conclude, these results demonstrate that peritoneal CD8^+^ T cells are hyperresponsive and exhibit cytokine-mediated bystander activation that correlates with markers of disease severity in patients with decompensated cirrhosis.

### Cytokine-mediated bystander activation of ascites CXCR6^+^CD69^+^ CD8^+^ T cells can be effectively blocked by the JAK inhibitor tofacitinib

**Fig. 7:**
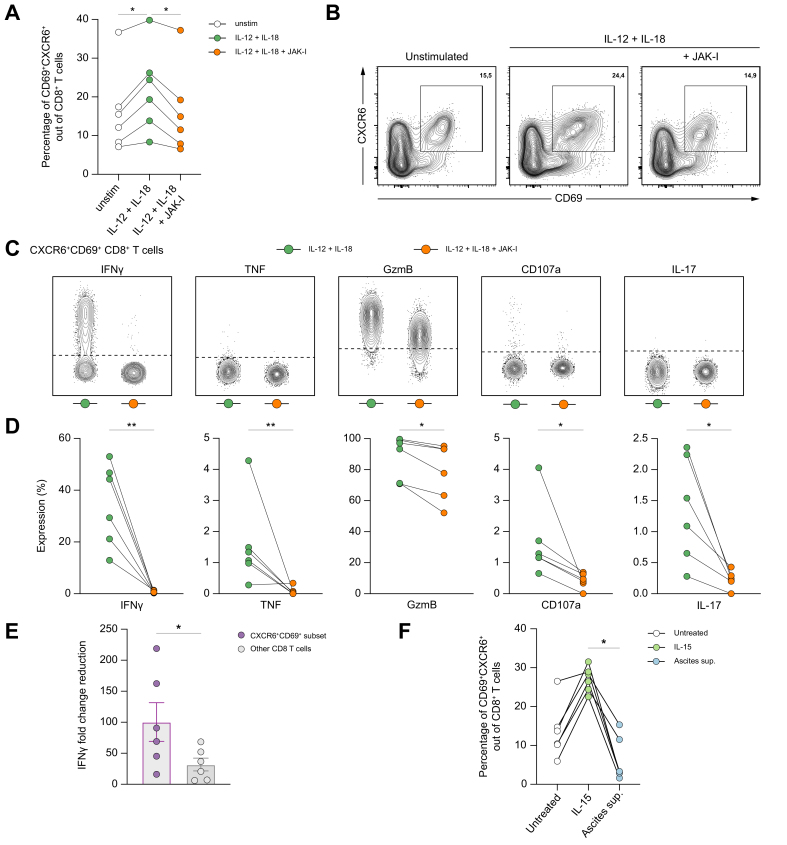
Cytokine-mediated bystander activation of ascites CXCR6^+^CD69^+^ CD8^+^ T cells can be effectively blocked by the JAK inhibitor tofacitinib. Experimental setup: *In vitro* treatment with the JAK inhibitor tofacitinib was performed on six ascites samples from patients with decompensated cirrhosis. (A) Frequencies of CXCR6^+^CD69^+^ CD8^+^ T cells out of total CD8^+^ T cells after stimulation with IL-12 + IL-18 in the presence (orange) or absence (green) of the JAK inhibitor tofacitinib compared with unstimulated medium controls (n = 6). (B) Representative FACS plots of one ascites sample showing CXCR6^+^CD69^+^ CD8^+^ T cells after stimulation with IL-12 + IL-18 ± JAK inhibitor compared with unstimulated medium control. (C, D) Representative concatenated FACS plots showing functional responses of ascites CXCR6^+^CD69^+^ CD8^+^ T cells after stimulation with IL-12 + IL-18 ± JAK inhibitor (C) and summary of the functional readouts (D). (E) IFNγ fold change reduction of CXCR6^+^CD69^+^ CD8^+^ T cells compared with their CD8^+^ T cell counterparts (CXCR6^+^CD69^+^ subset excluded) in the ascites. (F) Frequency of CXCR6^+^CD69^+^ CD8^+^ T cells in peripheral blood after co-incubation with IL-15 or ascites supernatant for 5 days. The Wilcoxon test or one-way ANOVA was used to determine statistical significance. ∗*p* <0.05; ∗∗*p* <0.01. GzmB, granzyme B; IFNγ, interferon γ; JAK, Janus kinase; PBMC, peripheral blood mononuclear cell; TNF, tumor necrosis factor.

In particular, IL-12, IL-15, and IL-18 are known to be potent stimulators that induce TCR-independent bystander activation of CD8^+^ T cells.^20^ Indeed, we observed a shift in phenotype toward higher frequencies of ascites CXCR6^+^CD69^+^ CD8^+^ T cells upon stimulation with IL-12 + IL-18 (Fig. 7A and B). Interestingly, this effect could be blocked by co-stimulation with the Janus kinase (JAK) inhibitor tofacitinib (Fig. 7A and B). In more depth, the JAK inhibitor tofacitinib was able to effectively block bystander-activated CXCR6^+^CD69^+^ CD8^+^ T cells and significantly suppress effector molecule production (Fig. 7C and D). Noteworthy, this effect was more pronounced in the subset of CXCR6^+^CD69^+^ CD8^+^ T cells than in CD8^+^ T cells that did not exhibit this phenotype, as evidenced by a higher IFNγ fold-change reduction (Fig. 7E). Of note, stimulation with IL-6 + IL-21 was also able to induce a CXCR6^+^CD69^+^ bystander phenotype in ascites, but these cytokine combinations did not result in functional responses (Fig. S4E).

Subsequently, we asked whether the accumulation of CXCR6^+^CD69^+^ CD8^+^ T cells in the ascites compared with blood could also be induced by the ascites milieu in the peritoneal cavity of patients with cirrhosis. To study this, PBMCs were co-cultured with ascites supernatant or IL-15 for 5 days in selected experiments. Interestingly, IL-15 stimulation resulted in a higher frequency of CXCR6^+^CD69^+^ cells in peripheral blood CD8^+^ T cells, whereas co-incubation with ascites supernatant did not result in an upregulation of CXCR6^+^CD69^+^ expression within CD8^+^ T cells (Fig. 7F). In line with this, co-incubation with ascites supernatant had no effect on the phenotype of blood CD8^+^ T cells, whereas IL-15 led to the upregulation of NKG2D, GzmB, and Perforin, markers related to bystander activation^20^ (Fig. S4F).

Moreover, as we have previously shown that ascites from patients with decompensated cirrhosis contains a pro-inflammatory cytokine milieu,^25^ together with our observations that IL-12, IL-15, and IL-18 were able to induce a bystander phenotype of CD8^+^ T cells, we investigated whether these cytokine levels correlated with the frequency of CXCR6^+^CD69^+^ CD8^+^ T cells. However, only a trend was observed for IL-12 correlating with the frequencies of CXCR6^+^CD69^+^ CD8^+^ T cells (Fig. S5A and B).

Taken together, these results demonstrate that the JAK inhibitor tofacitinib can effectively block cytokine-mediated bystander activation of CXCR6^+^CD69^+^ CD8^+^ T cells.

## Discussion

This study provides a comprehensive analysis of the CD8^+^ T-cell compartment in patients with decompensated cirrhosis and ascites. In more detail, in line with previous reports,^11^^,^^18^ we found that CD4^+^ as well as CD8^+^ T cells were decreased in the blood of patients with decompensated cirrhosis compared with healthy controls. Of note, CD8^+^ T cells were enriched in the ascites of patients with decompensated cirrhosis, whereas CD4^+^ T cells were decreased. The CD8^+^ T cells enriched in ascites showed an activated phenotype with high expression of tissue-retention markers. Remarkably, we detected several ascites-specific clusters characterized by high expression of CXCR6 and CD69, which were rarely found in blood. Co-expression of these markers was paralleled by high expression of NK cell receptors and activation/exhaustion markers, indicating a chronically activated late effector memory phenotype of CD8^+^ T cells in the peritoneal cavity. Together with the high responsiveness to cytokine-mediated activation by IL-12, IL-15, and IL-18, this indicates bystander activation of CXCR6^+^CD69^+^ CD8^+^ T cells. In line with this, we observed an upregulation of NKG2D and CCR5 on CD8^+^ T cells, which has been shown to be only evident in the absence of TCR stimulation.^20^^,^^41^ This was further corroborated by single-cell transcriptomics, which revealed an upregulation of several genes related to tissue residency and inflammation on CXCR6^+^CD69^+^ CD8^+^ T cells, as well as higher IL receptors and genes associated with effector function and pro-inflammatory cytokine production.

Recently, CXCR6^+^CD8^+^ T cells in the liver were annotated as auto-aggressive, contributing substantially to the immunopathogenesis of metabolic dysfunction-associated steatohepatitis.^12^ Indeed, we could demonstrate that frequencies of ascites CXCR6^+^CD69^+^ CD8^+^ T cells correlate with markers of disease severity in patients with decompensated cirrhosis, suggesting that these cells may play an important role in the disease pathogenesis. Furthermore, it is of utmost importance to understand the accumulation of CXCR6^+^CD69^+^ CD8^+^ bystander T cells in the peritoneal cavity and to elucidate whether these cells migrate into the ascites from other organs or whether this phenotype is induced by the ascites milieu present in the peritoneal cavity of patients with cirrhosis. Here, we show that mimicking the ascites milieu *in vitro* does not induce the bystander phenotype in circulating CD8^+^ T cells, and it is tempting to speculate that in patients with decompensated cirrhosis, CXCR6^+^CD69^+^ CD8^+^ T cells migrate from the liver into the ascites, where they reflect the degree of liver injury. Moreover, several recently published studies have demonstrated that overwhelming bystander activation of CD8^+^ T cells can lead to an inflammatory state and contribute to a fulminant course of acute infection,^15^ as well as directly drive liver damage through an innate-like hepatotoxic killing activity.[12], [13], [14]^,^^42^ Interestingly, we observed higher frequencies of ascites CXCR6^+^CD69^+^ CD8^+^ T cells in patients with ACLF in the absence of ongoing infection. This suggests that these cells, through their pro-inflammatory phenotype, may contribute to the exaggerated immune response that subsequently leads to systemic hyperinflammation, which is known to be the main cause of ACLF with consecutive (multiple) organ failure.^43^^,^^44^ This was further corroborated by correlations of IFNγ production of peritoneal CD8^+^ T cells and peritoneal CXCR6^+^CD69^+^ CD8^+^ T cells with markers of liver disease severity and renal impairment. These results align with previously published studies that showed that CD8^+^ T cells drive hyperinflammation in different chronic inflammatory diseases[45], [46], [47], [48], [49] and open up IFNγ-producing bystander CD8^+^ T cells as a possible targetable hallmark to suppress hyperinflammation. In contrast, during ongoing infection, bystander activation of CD8^+^ T cells may also have implications for host protection, including the early control of infecting pathogens, as they are activated well before the antigen-specific response is seen and are equipped with the ability to migrate to the site of infection.^50^

As it has previously been shown that in cytokine-mediated activation of CD8^+^ T cells, the JAK–STAT signaling route is responsible for IFNγ production,^20^^,^^45^^,^^51^ together with our observation that transcriptional data revealed an upregulation of the JAK–STAT pathway in CXCR6^+^CD69^+^ CD8^+^ T cells, we next investigated whether tofacitinib, a JAK inhibitor that primarily inhibits JAK1, JAK2, and JAK3,^52^ is able to block bystander-activated CD8^+^ T cells. Remarkably, tofacitinib not only was able to completely inhibit the phenotype shift toward CXCR6^+^CD69^+^ CD8^+^ T cells upon stimulation with IL-12 + IL-18, but was also able to suppress effector molecule production. As this effect was more pronounced within the bystander CXCR6^+^CD69^+^ CD8^+^ T cell compartment, we hypothesize that abrogation of the overshooting bystander T cell response is, in principle, possible. Further research should elaborate on strategies to target hyperinflammation in patients with decompensated cirrhosis. In line with this, recent studies suggest immunomodulation as a new approach to reduce inflammation and treat ACLF.^53^^,^^54^

The use of JAK inhibitors has been recently established in severe COVID-19 to suppress hyperinflammation and lung injury.[55], [56], [57] In this regard, anti-infective therapy is the main focus in the early stages of infection. However, thereafter, the treatment of hyperinflammation becomes of great importance.^58^ Moreover, it has been recently shown that JAK inhibitors ameliorate immune-mediated liver injury in mice^59^ and were able to attenuate fibrosis progression as well as accelerate fibrosis reversal.^60^^,^^61^ Nevertheless, there is an increased risk of infectious adverse events during treatment with JAK inhibitors.^62^

This study has some limitations. First, the heterogeneous patient cohort comprising different cirrhosis etiologies and the small number of patients with ACLF may have obscured certain group differences. Second, tissue-resident memory T cells and innate T cells, including MAIT cells, ɣδ T cells, and NKT cells, share common functional characteristics such as cytokine production, cytotoxic activity, and activation by TCR-independent mechanisms. Therefore, in principle, the possibility of contamination of the CXCR6^+^CD69^+^ CD8^+^ T cell subset by these cells exists.^30^ To address these concerns, we included MAIT cells from one patient in our transcriptomic analysis and found significant transcriptional and TCR diversity differences (Fig. S7B). In addition, the frequencies of MAIT cells as well as ɣδ T cells and NKT cells were very low in the CXCR6^+^CD69^+^ CD8^+^ T-cell subset (Fig. S4B). Furthermore, the role of TCR-mediated signaling in the activation of CXCR6^+^CD69^+^ CD8^+^ T cells cannot be completely excluded.

In conclusion, this study demonstrates that bystander-activated CXCR6^+^CD69^+^ CD8^+^ T cells are abundant in the ascites of patients with decompensated cirrhosis and are associated with liver disease severity. Interestingly, the JAK inhibitor tofacitinib can effectively block bystander CXCR6^+^CD69^+^ CD8^+^ T cells *in vitro*. Further studies are needed to evaluate how targeted inhibition of hyperinflammation could be an appropriate treatment option for patients with decompensated cirrhosis.

## Financial support

This work was funded by the 10.13039/100009139German Center for Infection Research (DZIF; TTU-05-701, TTU-05-708, TTU-05-711, and TTU-IICH-07-808). MC and HW were funded by the Deutsche Forschungsgemeinschaft (DFG, German Research Foundation) under Germany's Excellence Strategy – EXC 2155 – project number 390874280. The INFEKTA registry (DRKS00010664) was supported by the HepNet Study House of the German Liver Foundation. CAIMed was funded by the 10.13039/501100010570Ministry for Science and Culture of Lower Saxony with funds from the program “zukunft.niedersachsen” of the VolkswagenStiftung.

## Authors’ contributions

Study design: CN, NB, ARM, MC. Patient recruitment: MC, BM, HW. Experiments: CN, BS, EF, SK. Data analysis and statistics: CN, SK. Drafting of the manuscript: CN, MC. Critical revision of the manuscript: all authors.

## Data availability statement

Data are available upon request.

## Conflicts of interest

All authors declare no conflicts of interest related to this work.

Please refer to the accompanying ICMJE disclosure forms for further details.

## References

[1] GBD 2017 Cirrhosis Collaborators (2020). The global, regional, and national burden of cirrhosis by cause in 195 countries and territories, 1990–2017: a systematic analysis for the Global Burden of Disease Study 2017. Lancet Gastroenterol Hepatol.

[2] Asrani S.K., Devarbhavi H., Eaton J. (2019). Burden of liver diseases in the world. J Hepatol.

[3] Karlsen T.H., Sheron N., Zelber-Sagi S. (2022). The EASL–*Lancet* Liver Commission: protecting the next generation of Europeans against liver disease complications and premature mortality. Lancet.

[4] Arvaniti V., D’Amico G., Fede G. (2010). Infections in patients with cirrhosis increase mortality four-fold and should be used in determining prognosis. Gastroenterology.

[5] Bernal W., Jalan R., Quaglia A. (2015). Acute-on-chronic liver failure. Lancet.

[6] Engelmann C., Clària J., Szabo G. (2021). Pathophysiology of decompensated cirrhosis: portal hypertension, circulatory dysfunction, inflammation, metabolism and mitochondrial dysfunction. J Hepatol.

[7] Tsochatzis E.A., Bosch J., Burroughs A.K. (2014). Liver cirrhosis. Lancet.

[8] European Association for the Study of the Liver (2018). EASL Clinical Practice Guidelines for the management of patients with decompensated cirrhosis. J Hepatol.

[9] Albillos A., Lario M., Alvarez-Mon M. (2014). Cirrhosis-associated immune dysfunction: distinctive features and clinical relevance. J Hepatol.

[10] Jalan R., Fernandez J., Wiest R. (2014). Bacterial infections in cirrhosis: a position statement based on the EASL Special Conference 2013. J Hepatol.

[11] Lebossé F., Gudd C., Tunc E. (2019). CD8^+^T cells from patients with cirrhosis display a phenotype that may contribute to cirrhosis-associated immune dysfunction. EBioMedicine.

[12] Dudek M., Pfister D., Donakonda S. (2021). Auto-aggressive CXCR6^+^ CD8 T cells cause liver immune pathology in NASH. Nature.

[13] Kefalakes H., Horgan X.J., Jung M.K. (2021). Liver-resident bystander CD8^+^ T cells contribute to liver disease pathogenesis in chronic hepatitis D virus infection. Gastroenterology.

[14] Nkongolo S., Mahamed D., Kuipery A. (2023). Longitudinal liver sampling in patients with chronic hepatitis B starting antiviral therapy reveals hepatotoxic CD8^+^ T cells. J Clin Invest.

[15] Kim J., Chang D.Y., Lee H.W. (2018). Innate-like cytotoxic function of bystander-activated CD8^+^ T cells is associated with liver injury in acute hepatitis A. Immunity.

[16] Huang C.H., Fan J.H., Jeng W.J. (2022). Innate-like bystander-activated CD38^+^ HLA-DR^+^ CD8^+^ T cells play a pathogenic role in patients with chronic hepatitis C. Hepatology.

[17] You Z., Li Y., Wang Q. (2021). The clinical significance of hepatic CD69^+^ CD103^+^ CD8^+^ resident-memory T cells in autoimmune hepatitis. Hepatology.

[18] Rueschenbaum S., Ciesek S., Queck A. (2020). Dysregulated adaptive immunity is an early event in liver cirrhosis preceding acute-on-chronic liver failure. Front Immunol.

[19] Zhang N., Bevan M.J. (2011). CD8^+^ T cells: foot soldiers of the immune system. Immunity.

[20] Lee H., Jeong S., Shin E.-C. (2022). Significance of bystander T cell activation in microbial infection. Nat Immunol.

[21] Pallett L.J., Maini M.K. (2022). Liver-resident memory T cells: life in lockdown. Semin Immunopathol.

[22] Pallett L.J., Swadling L., Diniz M. (2023). Tissue CD14^+^CD8^+^ T cells reprogrammed by myeloid cells and modulated by LPS. Nature.

[23] Kim T.S., Shin E.C. (2019). The activation of bystander CD8^+^ T cells and their roles in viral infection. Exp Mol Med.

[24] Chapin C.A., Burn T., Meijome T. (2018). Indeterminate pediatric acute liver failure is uniquely characterized by a CD103^+^ CD8^+^ T-cell infiltrate. Hepatology.

[25] Niehaus C.E., Strunz B., Cornillet M. (2020). MAIT cells are enriched and highly functional in ascites of patients with decompensated liver cirrhosis. Hepatology.

[26] Moreau R., Jalan R., Gines P. (2013). Acute-on-chronic liver failure is a distinct syndrome that develops in patients with acute decompensation of cirrhosis. Gastroenterology.

[27] Hernaez R., Sola E., Moreau R. (2017). Acute-on-chronic liver failure: an update. Gut.

[28] Gerbes A.L., Gulberg V., Sauerbruch T. (2011). [German S 3-guideline “ascites, spontaneous bacterial peritonitis, hepatorenal syndrome”]. Z Gastroenterol.

[29] Bjorkstrom N.K., Fauriat C., Bryceson Y.T. (2010). Analysis of the KIR repertoire in human NK cells by flow cytometry. Methods Mol Biol.

[30] Ibidapo-Obe O., Bruns T. (2023). Tissue-resident and innate-like T cells in patients with advanced chronic liver disease. JHEP Rep.

[31] Becht E., McInnes L., Healy J. (2019). Dimensionality reduction for visualizing single-cell data using UMAP. Nat Biotechnol.

[32] Levine J.H., Simonds E.F., Bendall S.C. (2015). Data-driven phenotypic dissection of AML reveals progenitor-like cells that correlate with prognosis. Cell.

[33] Satija R., Farrell J.A., Gennert D. (2015). Spatial reconstruction of single-cell gene expression data. Nat Biotechnol.

[34] Wolf F.A., Angerer P., Theis F.J. (2018). SCANPY: large-scale single-cell gene expression data analysis. Genome Biol.

[35] Sturm G., Szabo T., Fotakis G. (2020). Scirpy: a Scanpy extension for analyzing single-cell T-cell receptor-sequencing data. Bioinformatics.

[36] Vivier E., Anfossi N. (2004). Inhibitory NK-cell receptors on T cells: witness of the past, actors of the future. Nat Rev Immunol.

[37] White J.T., Cross E.W., Burchill M.A. (2016). Virtual memory T cells develop and mediate bystander protective immunity in an IL-15-dependent manner. Nat Commun.

[38] Skon C.N., Lee J.Y., Anderson K.G. (2013). Transcriptional downregulation of *S1pr1* is required for the establishment of resident memory CD8^+^ T cells. Nat Immunol.

[39] Kumar B.V., Ma W., Miron M. (2017). Human tissue-resident memory T cells are defined by core transcriptional and functional signatures in lymphoid and mucosal Sites. Cell Rep.

[40] Borràs D.M., Verbandt S., Ausserhofer M. (2023). Single cell dynamics of tumor specificity vs bystander activity in CD8^+^ T cells define the diverse immune landscapes in colorectal cancer. Cell Discov.

[41] Seo I.H., Eun H.S., Kim J.K. (2021). IL-15 enhances CCR5-mediated migration of memory CD8^+^ T cells by upregulating CCR5 expression in the absence of TCR stimulation. Cel Rep.

[42] Luxenburger H., Neumann-Haefelin C. (2023). Liver-resident CD8^+^ T cells in viral hepatitis: not always good guys. J Clin Invest.

[43] Clària J., Stauber R.E., Coenraad M.J. (2016). Systemic inflammation in decompensated cirrhosis: characterization and role in acute-on-chronic liver failure. Hepatology.

[44] Zanetto A., Pelizzaro F., Campello E. (2023). Severity of systemic inflammation is the main predictor of ACLF and bleeding in individuals with acutely decompensated cirrhosis. J Hepatol.

[45] Sasson S.C., Slevin S.M., Cheung V.T.F. (2021). Interferon-gamma-producing CD8^+^ tissue resident memory T cells are a targetable hallmark of immune checkpoint inhibitor-colitis. Gastroenterology.

[46] Kopitar-Jerala N. (2017). The role of interferons in inflammation and inflammasome activation. Front Immunol.

[47] Roy E.R., Wang B., Wan Y.-W. (2020). Type I interferon response drives neuroinflammation and synapse loss in Alzheimer disease. J Clin Invest.

[48] Langer V., Vivi E., Regensburger D. (2019). IFN-γ drives inflammatory bowel disease pathogenesis through VE-cadherin–directed vascular barrier disruption. J Clin Invest.

[49] Bergamaschi L., Mescia F., Turner L. (2021). Longitudinal analysis reveals that delayed bystander CD8+ T cell activation and early immune pathology distinguish severe COVID-19 from mild disease. Immunity.

[50] Berg R.E., Crossley E., Murray S. (2003). Memory CD8^+^ T cells provide innate immune protection against *Listeria monocytogenes* in the absence of cognate antigen. J Exp Med.

[51] Novais F.O., Nguyen B.T., Scott P. (2021). Granzyme B inhibition by tofacitinib blocks the pathology induced by CD8 T cells in cutaneous leishmaniasis. J Invest Dermatol.

[52] Tanaka Y., Luo Y., O’Shea J.J. (2022). Janus kinase-targeting therapies in rheumatology: a mechanisms-based approach. Nat Rev Rheumatol.

[53] Engelmann C., Habtesion A., Hassan M. (2022). Combination of G-CSF and a TLR4 inhibitor reduce inflammation and promote regeneration in a mouse model of ACLF. J Hepatol.

[54] Engelmann C., Herber A., Franke A. (2021). Granulocyte-colony stimulating factor (G-CSF) to treat acute-on-chronic liver failure: a multicenter randomized trial (GRAFT study). J Hepatol.

[55] Guimarães P.O., Quirk D., Furtado R.H. (2021). Tofacitinib in patients hospitalized with Covid-19 pneumonia. N Engl J Med.

[56] Kalil A.C., Patterson T.F., Mehta A.K. (2021). Baricitinib plus remdesivir for hospitalized adults with Covid-19. N Engl J Med.

[57] Gustine J.N., Jones D. (2021). Immunopathology of hyperinflammation in COVID-19. Am J Pathol.

[58] Spinelli F.R., Conti F., Gadina M. (2020). HiJAKing SARS-CoV-2? The potential role of JAK inhibitors in the management of COVID-19. Sci Immunol.

[59] Wang H., Feng X., Han P. (2019). The JAK inhibitor tofacitinib ameliorates immune-mediated liver injury in mice. Mol Med Rep.

[60] Torres S., Ortiz C., Bachtler N. (2023). Janus kinase 2 inhibition by pacritinib as potential therapeutic target for liver fibrosis. Hepatology.

[61] Song Z., Liu X., Zhang W. (2022). Ruxolitinib suppresses liver fibrosis progression and accelerates fibrosis reversal via selectively targeting Janus kinase 1/2. J Transl Med.

[62] Adas M.A., Alveyn E., Cook E. (2022). The infection risks of JAK inhibition. Expert Rev Clin Immunol.

