# Supplementary material for: CXCR6+CD69+ CD8+ T cells in ascites are associated with disease severity in patients with cirrhosis
Source: JHEP Rep. 2024 Mar 24;6(6):101074. doi: 10.1016/j.jhepr.2024.101074 (PMC11179582; doi:10.1016/j.jhepr.2024.101074)
Supplement: Multimedia component 1 [file mmc1.pdf]

CXCR6$^+$CD69$^+$ CD8$^+$ T cells in ascites are associated with disease severity in patients with cirrhosis

Christian Niehaus, Sebastian Klein, Benedikt Strunz, Erich Freyer, Benjamin Maasoumy, Heiner Wedemeyer, Niklas K. Björkström, Anke RM Kraft, Markus Cornberg

Table of contents

Fig. S1……………………………………………………………………………………2

Fig. S2……………………………………………………………………………………3

Fig. S3……………………………………………………………………………………4

Fig. S4……………………………………………………………………………………6

Fig. S5……………………………………………………………………………………8

Fig. S6……………………………………………………………………………………9

Fig. S7…………………………………………………………………………………..10

Table S1………………………………………………………………………………..11

Supplementary Fig. 1. Gating strategy and CXCR6$^+$CD69$^+$ CD8$^+$ T cells in patients with ACLF versus non-ACLF.

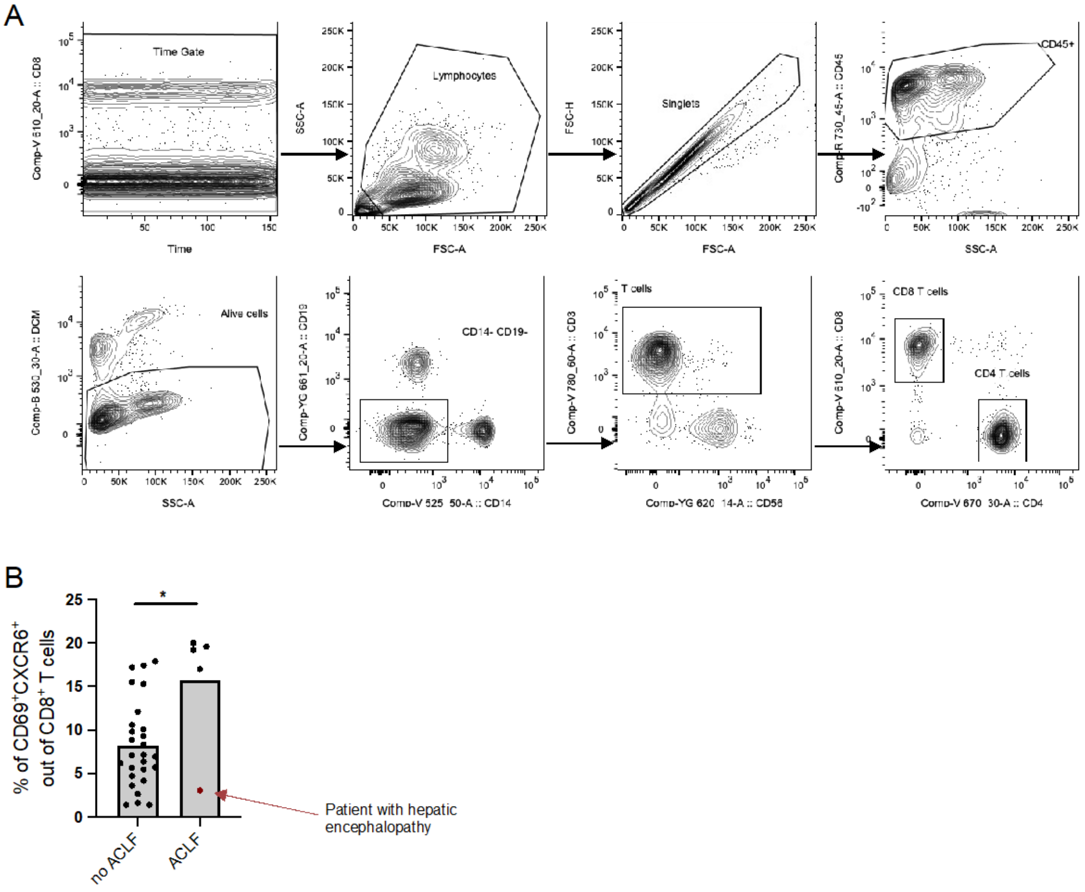

Fig. S1. Gating strategy and CXCR6$^+$CD69$^+$ CD8$^+$ T cells in patients with ACLF versus non-ACLF.

(A) To identify T cells, first a time gate was set to exclude clumps. This was followed by gating for lymphocytes and exclusion of doublet events followed by removal of CD14$^+$CD19$^+$ cells and dead cells. Thereafter, gating for CD3$^+$ cells was performed to identify T cells and subsequently gating for CD8 and CD4 to identify CD8$^+$ and CD4$^+$ T cells, respectively. (B) Frequencies of ascites CXCR6$^+$CD69$^+$ CD8$^+$ T cells in patients with decompensated liver cirrhosis and ACLF (n=27) compared to non-ACLF (n=5) patients. Mann-Whitney test was performed to determine statistical significance. * $p<0.05$.

Supplementary Fig. 2. Activation and exhaustion of CD8^{+} T cells in the peritoneal cavity.

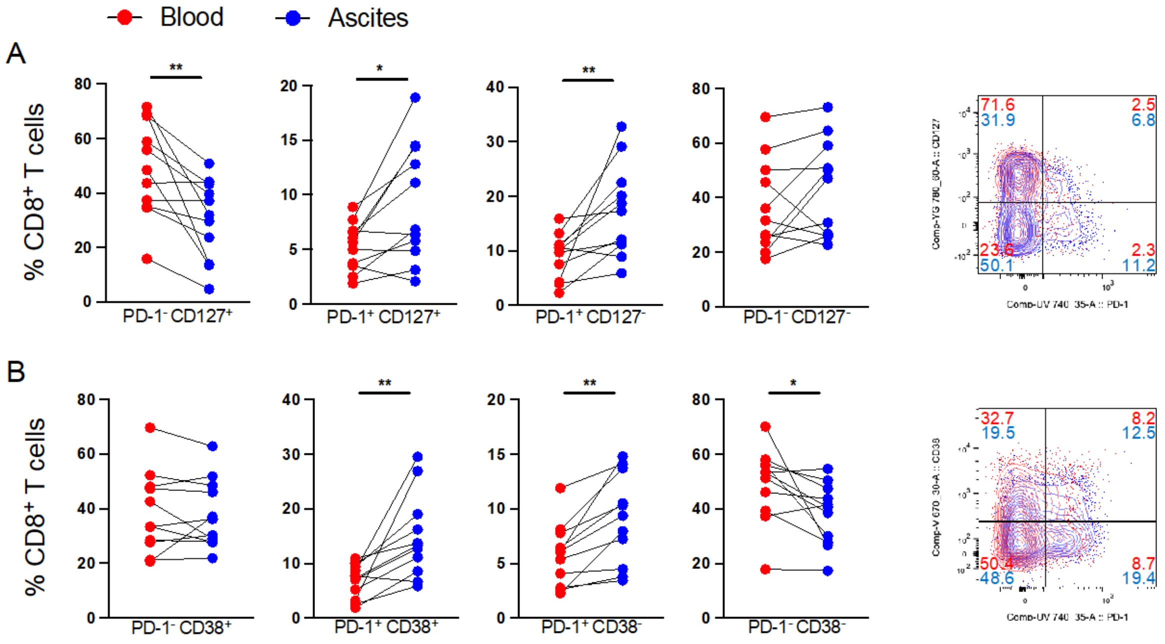

Fig. S2. Activation and exhaustion of CD8^{+} T cells in the peritoneal cavity.

(A,B) Coexpression of PD-1 and CD127 (A) and PD-1 and CD38, respectively (B) on matched blood and ascites CD8^{+} T cells (n=11) and representative staining of matched blood and ascites from one patient depicted as overlay (blood - red; ascites - blue). Comparisons for indicated phenotypic markers were done using paired *t* test. * $p<0.05$; ** $p<0.01$.

Supplementary Fig. 3. Phenotype of CD8+ T cells in the peritoneal cavity.

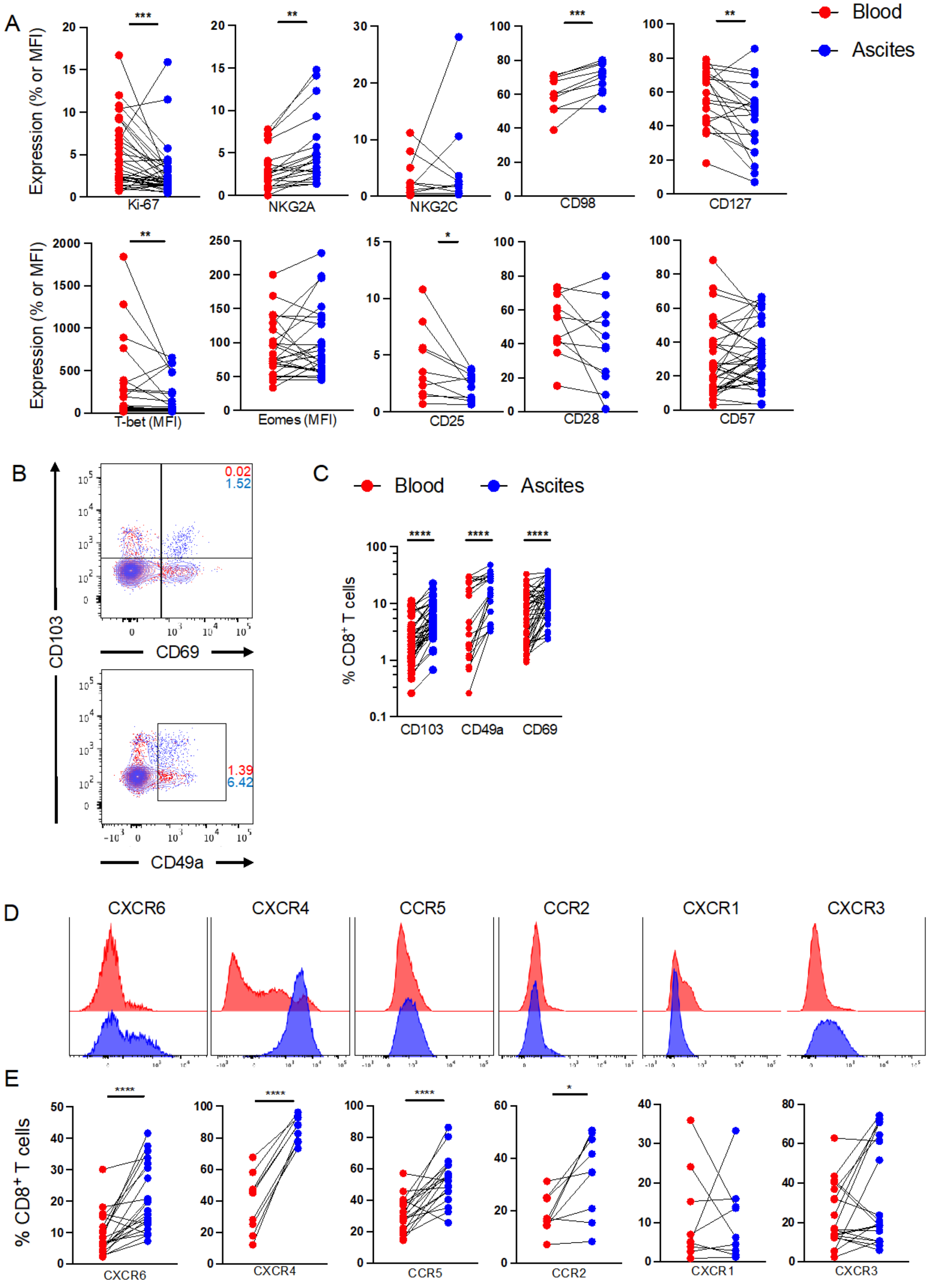

Fig. S3. Phenotype of $CD8^+$ T cells in the peritoneal cavity.

(A) Comparisons of indicated phenotypic markers were performed on matched blood and ascites samples (n=10-37). (B,C) Representative staining of tissue-residency marker expression on $CD8^+$ T cells in matched blood and ascites depicted as overlay (blood - red; ascites - blue) (B), and frequencies of these markers in blood compared to matched ascites (n=21-43) (C). (D,E) Expression of various tissue-residency markers on $CD8^+$ T cells from matched blood and ascites samples (n=9-21) (E), and the respective histograms from concatenated $CD8^+$ T cells (D). Paired *t* test or Wilcoxon test was used to determine statistical significance. * $p<0.05$; ** $p<0.01$; *** $p<0.001$; **** $p<0.0001$.

Supplementary Fig. 4. Functional analysis of CD8⁺ T cells in the peritoneal cavity.

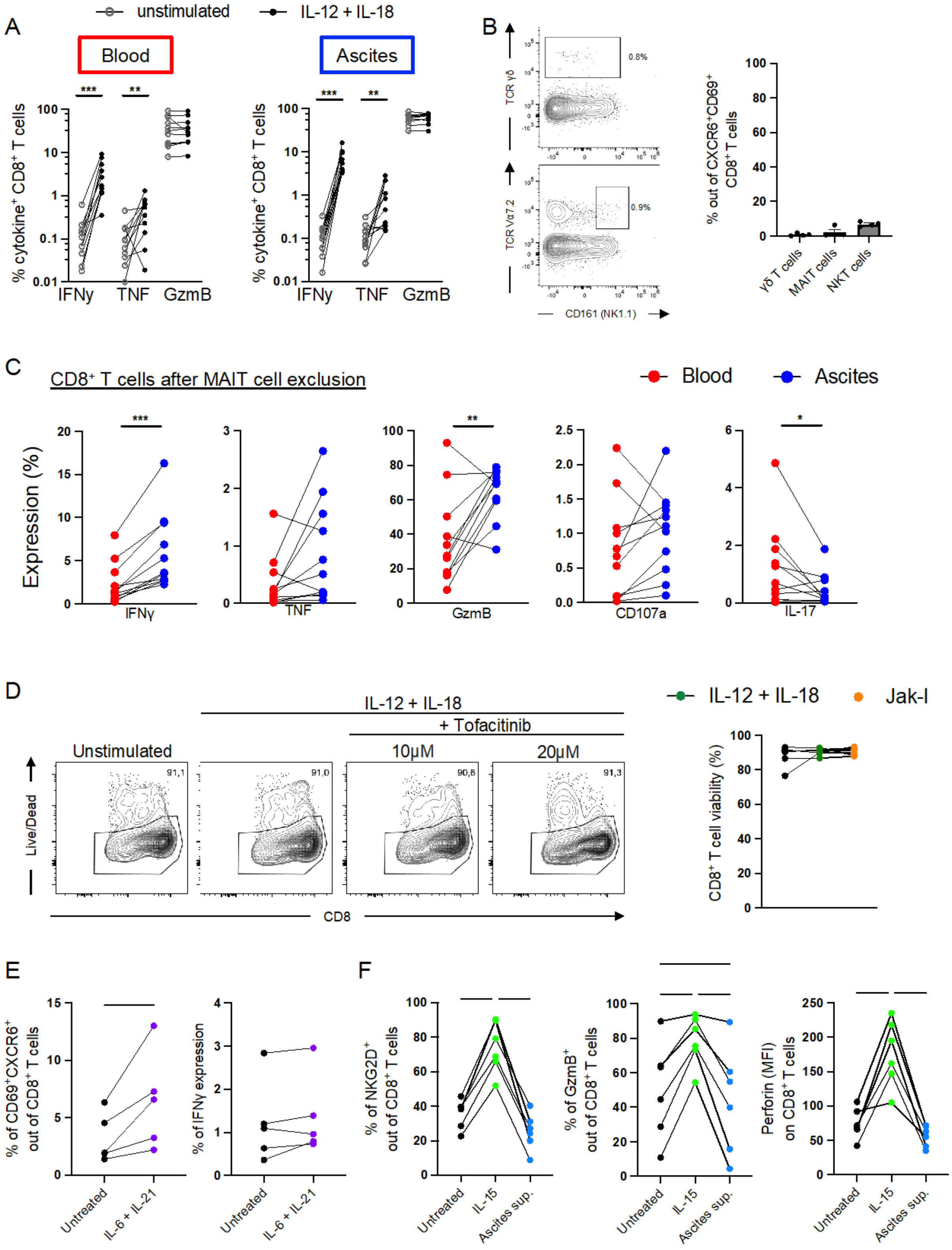

Fig. S4. Functional analysis of $CD8^+$ T cells in the peritoneal cavity.

(A) Production of effector molecules in blood (left) and ascites (right) $CD8^+$ T cells after stimulation with IL-12 + IL-18 compared to untreated medium controls (n=11). (B) Expression of indicated effector molecules after stimulation with IL-12 + IL-18 in matched blood and ascites $CD8^+$ T cells after MAIT cell exclusion (n=11). (C) $CD8^+$ T cell viability after stimulation with IL-12 + IL-18 ± Tofacitinib and representative FACS plots (n=6). (D) $CXCR6^+CD69^+$ $CD8^+$ T cell frequencies and frequencies of IFNγ-producing $CXCR6^+CD69^+$ $CD8^+$ T cells in the ascites after stimulation with IL-6 + IL-21 compared with unstimulated medium controls (n=5). (E) Frequency or MFI of indicated markers associated with bystander-activation of $CD8^+$ T cells in peripheral blood after coincubation with IL-15 or ascites supernatant for five days. Mann-Whitney test, unpaired *t*-test, Wilcoxon test, paired *t*-test or one-way ANOVA was used when appropriate. * $p<0.05$; ** $p<0.01$; *** $p<0.001$.

Supplementary Fig. 5. Correlations of cytokine values with frequencies of CXCR6⁺CD69⁺ CD8⁺ T cells.

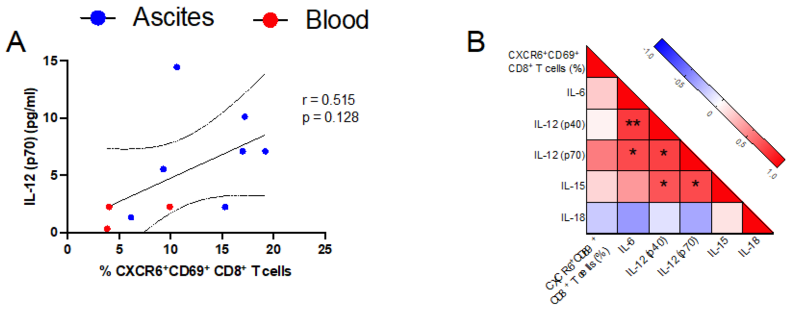

Fig. S5. Correlations of cytokine values with frequencies of CXCR6$^+$CD69$^+$ CD8$^+$ T cells.

(A) Correlation of IL-12 (p70) levels in blood and ascites with frequencies of CXCR6$^+$CD69$^+$ CD8$^+$ T cells from the same patients. (B) Correlation matrix of distinct cytokines with frequencies of CXCR6$^+$CD69$^+$ CD8$^+$ T cells. Correlations between cytokine values and CXCR6$^+$CD69$^+$ CD8$^+$ T cells were assumed using Spearman`s *r* or Pearson`s *r* when appropriate. * p<0.05; ** p<0.01.

Supplementary Fig. 6. Correlation matrix of clinical markers and T cell frequencies in blood and ascites.

A

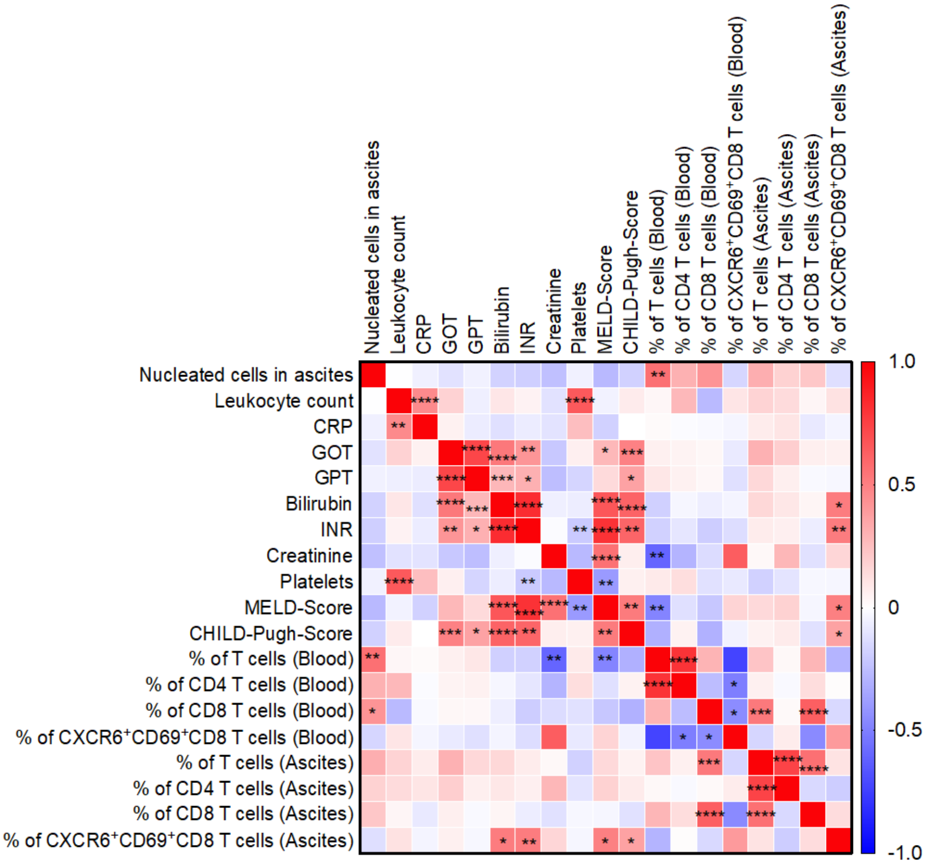

Fig. S6. Correlation matrix of clinical markers and T cell frequencies in blood and ascites.

(A) Correlation of relevant clinical markers of inflammation and liver disease severity with T cell frequencies and phenotype in blood and ascites. Correlations were assumed using Spearman`s *r* or Pearson`s *r* when appropriate. * $p<0.05$; ** $p<0.01$; *** $p<0.001$; **** $p<0.0001$.

Supplementary Fig. 7. TCR receptor analysis of CXCR6^{+}CD69^{+} CD8^{+} T cells compared with CD8^{+} T cells.

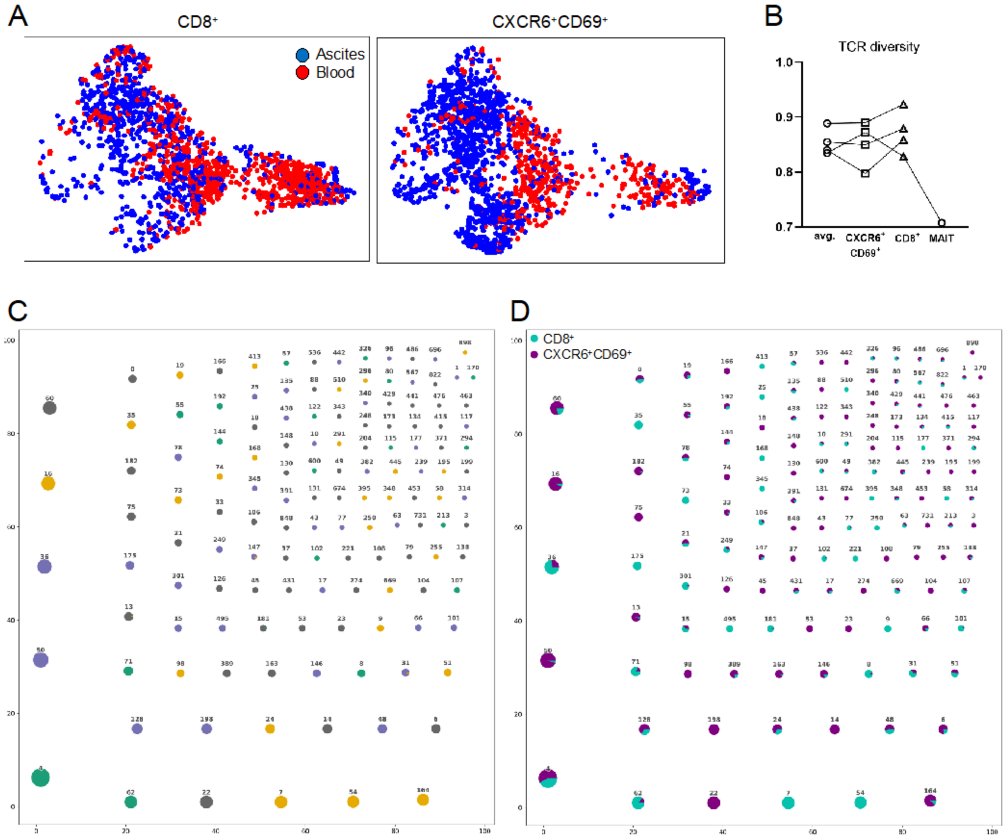

Fig. S7. TCR receptor analysis of CXCR6^{+}CD69^{+} CD8^{+} T cells compared with CD8^{+} T cells.

(A) UMAP of CXCR6^{+}CD69^{+} CD8^{+} and CXCR6^{-}CD69^{-} CD8^{+} T cells from a single patient, with colors indicating blood and ascites origin. (B) TCR diversity of all clonotypes compared between CXCR6^{+}CD69^{+} CD8^{+} T cells, total CXCR6^{-}CD69^{-} CD8^{+} T cells, and MAIT cells (from only one patient). (C) Pie chart of individual clonotypes in relation to their patients` origin (in %). Individual clonotypes are ordered from most abundant (lower left) to less abundant (upper right). Cutoff is set to clonotypes with at least 5 cells, less abundant clonotypes are not shown. Numbers refer to individual clonotypes and are consistent between C and D. (D) Same pie chart as depicted in C, except that the

frequencies of individual clonotypes appearing in either the $CXCR6^+CD69^+$ $CD8^+$ or the $CXCR6^-CD69^-$ $CD8^+$ compartment are shown.

Table S1. Patient characteristics of all patients included in this study.

Parameter	Healthy	Compensated cirrhosis	Decompensated cirrhosis	ACLF
**Number of patients**	24	10	Total: 50	5
**Gender**				
- Female	12	5	21	1
- Male	12	5	29	4
**Age**	58.1 ±7.8	59.5 ±14.8	62.8 ±8.8	67 ±6.6
**CHILD-Pugh Score (A/B/C)**	/	10/0/0	0/34/16	0/0/5
**MELD score**	/	6 ±1	15 ±9	21 ±13
**ACLF (grade 1/2/3)**	/	/	/	5/0/0
- Cause of ACLF: (AKI/HE/Coagulopathy)	/	/	/	3/1/1
**Antibiotic treatment within the last 3 months (y/n)**	/	0/10	11/38	2/5
**Antibiotic prophylaxis (y/n)**			5/45	0/5
**AST (U/L)**	/	88 ±60.6	52.4 ±30.8	50.4 ±32.9
**ALT (U/L)**	/	110.9 ±133.3	26.8 ±22.1	25 ±16.6
**Bilirubin (umol/L)**	/	12.9 ±6	48.3 ±81.1	78.8 ±72.7
**Albumin (g/dL)**	/	3.8 ±0.4	2.9 ±0.6	2.6 ±0.9
**INR**	/	1.2 ±0.2	1.4 ±0.4	1.7 ±0.7
**CRP (mg/L)**	/	4.4 ±3.6	29.9 ±36.1	20.9 ±16.4
**Etiology**				
- HCV	/	4	5	1
- HBV/HDV	/	6	4	0
- ARC	/	/	40	4

All values provided as mean ± SD
ACLF, acute-on-chronic liver failure; AKI, acute kidney injury; ALT, alanine transaminase; ARC, alcohol-related liver cirrhosis; AST, aspartate transaminase; CRP, C-reactive protein; HE, hepatic encephalopathy; INR, International Normalized Ratio; MELD, model of end stage liver disease.
