# Supplementary material for: CXCR6+CD69+ CD8+ T cells in ascites are associated with disease severity in patients with cirrhosis
Source: JHEP Rep. 2024 Mar 24;6(6):101074. doi: 10.1016/j.jhepr.2024.101074 (PMC11179582; doi:10.1016/j.jhepr.2024.101074)
Supplement: Multimedia component 2 [file mmc2.docx]

**Journal of Hepatology**

**CTAT methods**

Tables for a “Complete, Transparent, Accurate and Timely account” (CTAT) are now mandatory for all revised submissions. The aim is to enhance the reproducibility of methods.

- Only include the parts relevant to your study
- Refer to the CTAT in the main text as ‘Supplementary CTAT Table’
- Do not add subheadings
- Add as many rows as needed to include all information
- Only include one item per row

**If the CTAT form is not relevant to your study, please outline the reasons why:**

|  |
| --- |

- 1. **Antibodies**

| **Antibody name** | **Color** | **Clone** | **Supplier** |
| --- | --- | --- | --- |
| anti-Ki-67 | Alexa Fluor 488 | Ki-67 | BioLegend |
| anti-CD107a (LAMP-1) | Alexa Fluor 647 | H4A3 | BioLegend |
| anti-CD49a (Integrin α1) | Alexa Fluor 647 | TS2/7 | BioLegend |
| anti-CCR5 (CD195) | Alexa Fluor 647 | HEK/1/85a | BioLegend |
| anti-CD45 (LCA) | Alexa Fluor 700 | HI30 | BioLegend |
| anti-CD103 (Integrin αE) | APC | Ber-ACT8 | BioLegend |
| anti-CD57 | APC | QA17A04 | BioLegend |
| anti-HLA-DR | APC-Cy7 | L243 | BioLegend |
| anti-CD57 | Pacific Blue | HCD57 | BioLegend |
| anti-CD8 | APC-Fire750 | RPA-T8 | BioLegend |
| anti-CD14 | V500 | M5E2 | BioLegend |
| anti-CD279 (PD-1) | Brilliant Violet 421 | EH12.2H7 | BioLegend |
| anti-IFN-γ | Brilliant Violet 421 | 4S.B3 | BioLegend |
| anti-IFN-γ | Brilliant Violet 785 | 4S.B3 | BioLegend |
| anti-T-bet | Brilliant Violet 421 | 4B10 | BioLegend |
| anti-CXCR6 (CD186) | Brilliant Violet 421 | K041E5 | BioLegend |
| anti CD3 | Brilliant Violet 510 | UCHT1 | BioLegend |
| anti CD3 | Brilliant Violet 750 | 17A2 | BioLegend |
| anti-CD4 | Brilliant Violet 570 | OKT4 | BioLegend |
| anti-CD8 | Brilliant Violet 605 | 53-6.7 | BioLegend |
| anti-CD69 | Brilliant Violet 786 | FN50 | BioLegend |
| anti-CD161 | Brilliant Violet 605 | HP-3G10 | BioLegend |
| anti-CD161 | APC-Cy7 | HP-3G10 | BioLegend |
| anti-CD16 | Brilliant Violet 650 | 3G8 | BioLegend |
| anti-HLA-DR | Brilliant Violet 650 | L243 | BioLegend |
| anti-TNF-α | Brilliant Violet 650 | Mab11 | BioLegend |
| anti-CD38 | Brilliant Violet 650 | HB-7 | BioLegend |
| anti-CD14 | Brilliant Violet 711 | M5E2 | BioLegend |
| anti-CD19 | Brilliant Violet 711 | HIB19 | BioLegend |
| anti-CD19 | APC/Fire 810 | HIB19 | BioLegend |
| anti-CD25 | Brilliant Violet 711 | BC96 | BioLegend |
| anti-TCR Vα7.2 | Brilliant Violet 785 | 3C10 | BioLegend |
| anti-CD45RA | FITC | HI100 | BioLegend |
| anti-CD197 (CCR7) | PE | G043H7 | BioLegend |
| anti-Granzyme B | PE | QA16A02 | BioLegend |
| anti-TCR Vα7.2 | PE | 3C10 | BioLegend |
| anti-CD141 (Thrombomodulin) | PE | M80 | BioLegend |
| anti-CD3 | PE-Cy5 | UCHT1 | BioLegend |
| anti-CD19 | PE-Cy5 | HIB19 | BioLegend |
| anti-CXCR4 (CD184) | PE-Cy5 | 12G5 | BioLegend |
| anti-CD56 (NCAM) | PE-Cy7 | QA17A16 | BioLegend |
| anti-TCR Vα7.2 | PE-Cy7 | 3C10 | BioLegend |
| anti-CD127 | PE-Cy7 | A019D5 | BioLegend |
| anti-CD69 | PE-Dazzle594 | FN50 | BioLegend |
| anti-IL-17A | PE-Dazzle594 | BL168 | BioLegend |
| anti-CD4 | PerCP-Cy5.5 | RPA-T4 | BioLegend |
| anti-Bax | AlexaFluor488 | 2D2 | BioLegend |
| anti-CD197 (CCR7) | Brilliant Violet 421 | G043H7 | BioLegend |
| anti-BDCA-2 | Brilliant Violet 421 | 201A | BioLegend |
| anti-Bcl-2 | PE | Bcl-2/100 | BioLegend |
| anti-CD45RA | APC | HI100 | BD Biosciences |
| anti-CD45RA | Brilliant Violet 786 | HI100 | BD Biosciences |
| anti-NKp46 | Brilliant Violet 786 | 9E2/NKp46 | BD Biosciences |
| anti-CD103 (Integrin αE) | FITC | Ber-ACT8 | BD Biosciences |
| anti-CD103 (Integrin αE) | Brilliant Violet 711 | Ber-ACT8 | BD Biosciences |
| anti-CD103 (Integrin αE) | BB660 | Ber-ACT8 | BD Biosciences |
| anti-CD39 | Brilliant Violet 711 | A1 | BD Biosciences |
| anti-Perforin | BB755 | δG9 | BD Biosciences |
| anti-Granzyme B | BB790 | GB11 | BD Biosciences |
| anti-CD107a | FITC | H4A3 | BD Biosciences |
| anti-CD16 | APC-Cy7 | 3G8 | BD Biosciences |
| anti-CD163 | Alexa Fluor 647 | GHI/61 | BD Biosciences |
| Fixable Viability Stain 700 |  |  | BD Biosciences |
| anti-CXCR3 (CD183) | Brilliant Violet 421 | 1C6/CXCR3 | BD Biosciences |
| anti-CD3 | Brilliant Violet 786 | SK7 | BD Biosciences |
| anti-CD3 | Brilliant Ultraviolet661 | UCHT1 | BD Biosciences |
| anti-CD14 | Violet 500 | M5E2 | BD Biosciences |
| anti-CD19 | Brilliant Violet 510 | SJ24C1 | BD Biosciences |
| anti-CD98 | Brilliant Violet 650 | UM7F8 | BD Biosciences |
| anti-CD4 | PE-CF594 | RPA-T4 | BD Biosciences |
| anti-CD56 | PE-CF594 | NCAM 16.2 | BD Biosciences |
| anti-TNF-α | PE-CF594 | Mab11 | BD Biosciences |
| anti-CD56 | Brilliant Ultraviolet563 | NCAM 16.2 | BD Biosciences |
| anti-CD49a (Integrin α1) | Brilliant Ultraviolet615 | SR84 | BD Biosciences |
| anti-CD4 | Brilliant Ultraviolet737 | SK3 | BD Biosciences |
| anti-NKG2D | Brilliant Ultraviolet737 | 1D11 | BD Biosciences |
| anti-HLA-DR | Brilliant Ultraviolet395 | G46-6 | BD Biosciences |
| anti-CD16 | Brilliant Ultraviolet737 | 3G8 | BD Biosciences |
| anti-CD16 | Brilliant Ultraviolet496 | 3G8 | BD Biosciences |
| anti-CD69 | Brilliant Ultraviolet395 | FN50 | BD Biosciences |
| anti-CD69 | Brilliant Ultraviolet737 | FN50 | BD Biosciences |
| anti-TCR Vα7.2 | Brilliant Ultraviolet615 | OF-5A12 | BD Biosciences |
| anti-Ki-67 | Brilliant Ultraviolet395 | B56 | BD Biosciences |
| anti-CD279 (PD-1) | Brilliant Ultraviolet737 | EH12.1 | BD Biosciences |
| anti-CD8 | Brilliant Ultraviolet805 | SK1 | BD Biosciences |
| anti-CXCR3 | Brilliant Ultraviolet805 | 1C6/CXCR3 | BD Biosciences |
| anti-Eomes | PE-eFluor 610 | WD1928 | ThermoFisher |
| anti-CD4 | QD655 | S3.5 | ThermoFisher |
| anti-CD8 | QD605 | 3B5 | ThermoFisher |
| Streptavidin | QD585 |  | ThermoFisher |
| Live Dead Fixable Aqua |  |  | ThermoFisher |
| Live Dead Fixable Green |  |  | ThermoFisher |
| Live Dead Fixable Blue |  |  | ThermoFisher |
| anti-CD161 | Biotin | 191B8 | Miltenyi |
| anti-CD14 | APC | REA599 | Miltenyi |
| anti-TCRγδ | PE | 11F2 | Miltenyi |
| anti-CD49e | VioBright; FITC | REA686 | Miltenyi |
| anti-NKG2C | Biotin | REA205 | Miltenyi |
| anti-CD57 | APC-Vio770 | TB03 | Miltenyi |
| anti-CD28 | PE-Cy5.5 | CD28.2 | Beckman Coulter |
| anti-TCRγδ | PE-Cy5.5 | IMMU510 | Beckman Coulter |
| anti-CD117 (c-kit) | PE-Cy5.5 | 95C3 | Beckman Coulter |
| anti-CD56 | PE-Cy5.5 | N901 | Beckman Coulter |
| anti-KIR2DL1,  anti-KIR2DS1 | PE-Cy5.5 | EB6 | Beckman Coulter |
| anti-CD69 | ECD | TP1.55.3 | Beckman Coulter |
| anti-NKG2A (CD159a) | APC | Z199 | Beckman Coulter |

- 1. **Cell lines**

| **Name** | **Citation** | **Supplier** | **Cat no.** | **Passage no.** | **Authentication test method** |
| --- | --- | --- | --- | --- | --- |
| n.a. |  |  |  |  |  |

- 1. **Organisms**

| **Name** | **Citation** | **Supplier** | **Strain** | **Sex** | **Age** | **Overall n number** |
| --- | --- | --- | --- | --- | --- | --- |
| n.a. |  |  |  |  |  |  |

- 1. **Sequence based reagents**

| **Name** | **Sequence** | **Supplier** |
| --- | --- | --- |
| n.a. |  |  |

- 1. **Biological samples**

| **Description** | **Source** | **Identifier** |
| --- | --- | --- |
| Human peripheral blood | Healthy donors and cirrhosis patients |  |
| Human ascites fluid | Cirrhosis patients |  |

- 1. **Deposited data**

| **Name of repository** | **Identifier** | **Link** |
| --- | --- | --- |
| RNA-seq data will be made available upon request. |  |  |

- 1. **Software**

| **Software name** | **Manufacturer** | **Version** |
| --- | --- | --- |
| FlowJo software | BD Biosciences | 10.5.3 |
| Prism software | GraphPad | 9.0 |
| BD Rhapsody Analysis Pipeline | BD Biosciences | 1.9 |
| GNU R | R core team (open scource) | 4.2 |
| scanpy | Scanpy development team (open scource) |  |
| BioPlex Manager | Bio-Rad | 6.0 |

- 1. **Other (e.g. drugs, proteins, vectors etc.)**

| Tofacitinib | Sigma Aldrich |  |
| --- | --- | --- |

- 1. **Please provide the details of the corresponding methods author for the manuscript:**

| Markus Cornberg, Department of Gastroenterology, Hepatology, Infectious Diseases and Endocrinology, Hannover Medical School, Hannover, Germany.  E-mail: [Cornberg.Markus@mh-hannover.de](mailto:Cornberg.Markus@mh-hannover.de) |
| --- |

**2.0 Please confirm for randomised controlled trials all versions of the clinical protocol are included in the submission. These will be published online as supplementary information.**

| n.a. |
| --- |
